# A Functional Genomics Approach to Establish the Complement of Carbohydrate Transporters in *Streptococcus pneumoniae*

**DOI:** 10.1371/journal.pone.0033320

**Published:** 2012-03-13

**Authors:** Alessandro Bidossi, Laura Mulas, Francesca Decorosi, Leonarda Colomba, Susanna Ricci, Gianni Pozzi, Josef Deutscher, Carlo Viti, Marco Rinaldo Oggioni

**Affiliations:** 1 Lab. Microbiologia Molecolare e Biotecnologia, Dip. Biologia Molecolare, Università di Siena, Siena, Italy; 2 UOC Batteriologia, Azienda Ospedaliera Universitaria Senese, Siena, Italy; 3 Sezione Microbiologia, Dip. Biotecnologie Agrarie, Università degli Studi di Firenze, Firenze, Italy; 4 CNRS, CBAI, F-78850 Thiverval-Grignon, France; Instituto Butantan, Brazil

## Abstract

The aerotolerant anaerobe *Streptococcus pneumoniae* is part of the normal nasopharyngeal microbiota of humans and one of the most important invasive pathogens. A genomic survey allowed establishing the occurrence of twenty-one phosphotransferase systems, seven carbohydrate uptake ABC transporters, one sodium∶solute symporter and a permease, underlining an exceptionally high capacity for uptake of carbohydrate substrates. Despite high genomic variability, combined phenotypic and genomic analysis of twenty sequenced strains did assign the substrate specificity only to two uptake systems. Systematic analysis of mutants for most carbohydrate transporters enabled us to assign a phenotype and substrate specificity to twenty-three transport systems. For five putative transporters for galactose, pentoses, ribonucleosides and sulphated glycans activity was inferred, but not experimentally confirmed and only one transport system remains with an unknown substrate and lack of any functional annotation. Using a metabolic approach, 80% of the thirty-two fermentable carbon substrates were assigned to the corresponding transporter. The complexity and robustness of sugar uptake is underlined by the finding that many transporters have multiple substrates, and many sugars are transported by more than one system. The present work permits to draw a functional map of the complete arsenal of carbohydrate utilisation proteins of pneumococci, allows re-annotation of genomic data and might serve as a reference for related species. These data provide tools for specific investigation of the roles of the different carbon substrates on pneumococcal physiology in the host during carriage and invasive infection.

## Introduction

*Streptococcus pneumoniae*, the main cause of community acquired pneumonia, is one of the most important human pathogens and a frequent etiologic agent of sepsis, meningitis, otitis media and conjunctivitis [1]. Despite effective vaccines and appropriate antibiotic therapy the burden of invasive pneumococcal disease is still high worldwide, including both developed and developing countries [2], [3]. The colonisation of the human nasopharyngeal mucosa by *S. pneumoniae* is a natural process that occurs during the first few months of life. Successive episodes of colonisation are common and the duration of carriage episodes varies with a mean duration of one month depending on the serotype and the age of the infected individual [4], [5]. Most colonised individuals are asymptomatic, but occasionally progression towards disease occurs, generally early after acquisition of a new strain [4], [6], [7]. Both carriage and pneumococcal invasive disease show a clear seasonal variation with a peak in winter, which coincides with the seasonal peak of viral respiratory infections and indicates multi-variant environmental influences on host-microbe interaction and pathogenesis of disease [5], [8]. Recently we have shown that the nine carbon amino sugar sialic acid in saliva is a signal for pneumococcal virulence in the host providing a possible molecular explanation of the epidemiologic correlation between influenza and pneumococcal pneumonia [9].

**Figure 1:**
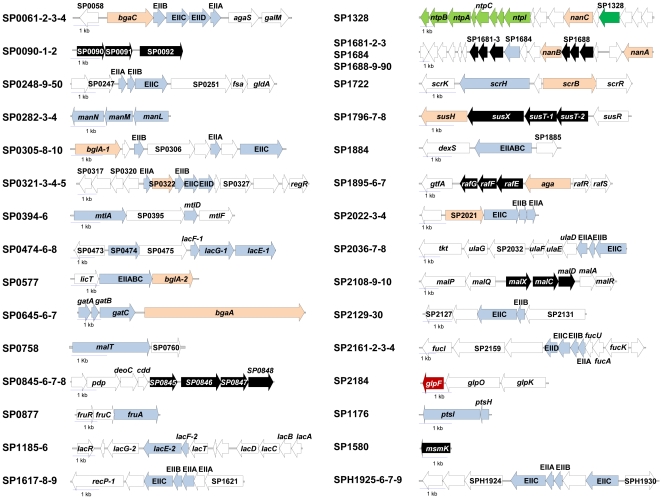
Schematic representation of all the genomic regions containing the pneumococcal sugar transporters. The genomic regions for the described 21 described PTS systems (PTS ORFs in light blue), the seven ABC transporters (black), the single sodium∶solute symporter (green) with downstream sodium ATPase (light green), the glycerol permease (red) of strain TIGR4 are shown. In addition to the substrate specific parts of the transporters of TIGR4 also the locus for the central PTS enzymes EI (SP1176 *ptsI*; light blue) and HpR (SP1177 *ptsH*; light blue) is shown, as also the monocystronic *msmK* gene encoding the ATP binding cassette protein energising some of the CUT1 permeases. The PTS transporter SPH1925-6-7, not present in TIGR4, is shown as in the genome of strain Hungary 19F.

Pneumococci are Gram-positive anaerobic bacteria with an exclusively fermentative metabolism for which textbooks and biochemical identification schemes list ten efficiently metabolised sugar substrates [10], [11]. This is somewhat in contrast with the high number of carbohydrate import systems, which are mainly primary active transporters belonging to the ATP-binding cassette (ABC) superfamily or the phosphotransfer-driven group translocators (PTS, phosphoenolpyruvate∶sugar phosphotransferase system) [12]–[15]. The most abundant group of carbohydrate transporters in the pneumococcus are the PTS transporters [15]. Each pneumococcal genome contains between 15 and 20 PTS transporters, with a total of 21 PTS systems identified in the collection of strains analysed (Tables 1 and 2). In the reference genome strain TIGR4 the PTS are classified, according to the transport classification scheme, in: (i) five transporters belonging to the TC 4.A.1. Glucose-Glucoside (Glc) Family (SP0577, SP0758, SP1684, SP1722, and SP1884), (ii) three to the 4.A.2 Fructose-Mannitol (Fru) Family (SP0394-6, SP0877 and SP1617-8-9), (iii) six to the 4.A.3 Lactose-N,N′-Diacetylchitobiose-β-glucoside (Lac) Family (SP0248-9-50, SP0305-8-10, SP0476-8, SP1185-6, SP2022-3-4 and SPH1925-6-7 in strain Hungary 19A), (iv) none to the 4.A.4 Glucitol (Gut) Family, (v) one to the 4.A.5 Galactitol (Gat) Family (SP0645-6-7), (vi) four to the 4.A.6 Mannose-Fructose-Sorbose (Man) Family (SP0061-2-3-4, SP0282-3-4, SP0321-3-4-5 and SP2161-2-3-4), and (vii) two to the 4.A.7 L-Ascorbate (L-Asc) Family (SP2036-7-8 and SP2129-30) [16], [17]. Most PTS systems are composed of an EIIA, EIIB and EIIC (EIID) domain in various arrangements [18], [19]. Exceptions are SP1684 (a PTS-Glc in the sialic acid operon) and SP2129-30 (a PTS-Asc), which are both missing an EIIA domain. EIIA devoid PTS systems have been described and shown in some cases to be cross-activated by EIIA-Glc [20], [21]. Two transporters SP0474-6-8 and SPH1925-6-7-9, which are both of the PTS-Lac family, have an additional gene for a EIIC domain in addition to those for EIIA, EIIB and EIIC. In order to perform a functional characterisation of the transporters we constructed mutants in all PTS systems of our reference recipient strain DP1004, a rough D39 derivative. A schematic representation of the 21 pneumococcal PTS transporters is given in Figure S1 and the map of the all chromosomal carbohydrate uptake related loci is given in Figure 1. The second most abundant group of sugar transport systems are ABC transporters. Carbohydrate uptake ABC transporters are grouped into two families CUT1 and CUT2, which differ for numerous characteristics including nature of substrates and structure [22]. In the pneumococcal genome there are six to seven CUT1 transporters and one CUT2 transporter. The CUT2 transporter (SP0846) is composed of two permeases, which form a heterodimer in the assembled transporter, one protein with two ATP binding cassette (ABC) domains and a substrate binding protein. All seven CUT1 transporters are composed of two permeases (heterodimer), one substrate binding protein, but are lacking the gene encoding the ABC subunit. In the pneumococcal genome there is only one “orphan” CUT1 ABC subunit-encoding gene (homodimeric protein) in a monocistronic operon (SP1580) [23]. In addition to the PTS and ABC transporter the pneumococcal genome encodes one sodium∶solute symporter and three aquaporin/glycerol-permeases which are possible carbohydrate uptake systems. Out of these thirty pneumococcal carbohydrate transport systems, so far only six were partially characterised, including the two PTS for beta-glucosides and sucrose and four ABC transporters for sialic acid, sucrose, maltose and raffinose [24]–[28].

**Table 1:** Pneumococcal carbohydrate uptake systems.

TIGR4	G54	R6	family	name	conservation^a^	Phenotype	substrates^b^	reference
SP0061-2-3-4	SPG00065-6-7-8	spr0060-1-2-3	PTS-Man		conserved		galactose	[99], [100]
SP0090-1-2	SPG00089-90-1	spr0081-2-3	CUT1		conserved	galactose, mannose, ManNAc		
SP0248-9-50	SPG00235-6-7	spr0229-30-1	PTS-Lac		conserved			
SP0282-3-4	SPG00265-6-7	spr0259-60-1	PTS-Man	*manLMN*	conserved	glucose, mannose, galactose, GlcNAc, GlcN	glucose, mannose, fructose, GlcNAc	[42]–[44]
SP0305-8-10	-	spr0278-80-82	PTS-Lac	*celBCD*	Indel^c^	cellobiose, gentiobiose, arbutin, amygdalin, aesculin	beta-glucosides	[26]
SP0321-3-4-5	SPG00291-3-4-5	spr0291-3-4-5	PTS-Man		conserved	hyaluronic acid	sulfated glycosaminoglycans	[49], [51]
SP0394-6	SPG00362-4	spr0356-8	PTS-Fru	*mtlAF*	indel	mannitol	mannitol	[54]
SP0474-6-8	SPG00432-4	spr0423-5	PTS-Lac		indel	mannose		
SP0577	SPG00525	spr0505	PTS-Glc	*bglP*	indel	1-O-Methyl-β-glucoside	beta-glucosides	[55]
SP0645-6-7	SPG00585-6-7	spr0562-3-4	PTS-Gat	*gatABC*	conserved	galactose	galactose, lactose, galactitol	[56]
SP0758	SPG00690	spr0668	PTS-Glc	*malT*	conserved	maltose, maltotriose, maltodextrin and glycogen	maltose	[58]–[60]
SP0845-6-7-8	SPG00765-6-7-8	spr0747-8-9-50	CUT2		conserved		ribonucleoside	[79]
SP0877	SPG00801	spr0780	PTS-Fru	*fruA*	conserved	fructose	fructose	[63]
SP1185-6	SPG01082-3	spr1070-1	PTS-Lac	*lacEF-2*	alleles	lactulose, lactose	lactose, tagatose	[65]–[68]
SP1328	SPG01221	spr1191-2-3-4	symporter	*ntp/app*	alleles	NeuNAc ManNAc	aminosugars	
SP1617-8-9	-	-	PTS-Fru		indel		pentoses	[70], [71]
SP1681-2-3	SPG01589-90-91	spr1525-6-7	CUT1		conserved	NeuNAc ManNAc	NeuNAc	[28], [72]
SP1684	SPG01592	spr1528	PTS-Glc		indel	GlcN		
SP1688-9-90	SPG01596-7-8	spr1532-3-4	CUT1		conserved	NeuNAc ManNAc	NeuNAc	[72]
SP1722	SPG01627	spr1566	PTS-Glc	*scrH*	alleles	sucrose	sucrose	[27]
SP1796-7-8	SPG01682-3-4	spr1618-9-20	CUT1	*susXT1T2*	alleles	sucrose	sucrose	[27]
-	SPG01697-8-701^d^	-	PTS-Lac		indel		sulphated glycans	[108]
SP1884	SPG01796	spr1699	PTS-Glc		indel	trehalose	trehalose	[73]
SP1895-6-7	SPG01782-3-4	spr1710-1-2	CUT1	*rafGFE*	conserved	stachyose, raffinose, melibiose	alpha-galactosides	
SP2022-3-4	SPG01935-6-7	spr1834-5-6	PTS-Lac		conserved	amygdalin	beta-glucosides	
SP2036-7-8	SPG01950-1-2	spr1847-8-9	PTS-Asc		conserved	ascorbate	ascorbate	[75], [76]
SP2108-9-10	SPG02045-6-7	spr1918-9-20	CUT1	*malXCD*	conserved	maltotriose, maltodextrin and glycogen	oligosaccharides	[25]
SP2129-30	SPG02066-7	spr1938-9	PTS-Asc		conserved		pentoses	
SP2161-2-3-4	SPG02105-6-7	spr1967-8-9-10	PTS-Man		alleles		fucose, L-arabinose	[77], [78]
SP2184	SPG02125	spr1988	facilitator	*glpF*	conserved	glycerol	glycerol	[89], [90]

(a) conservation as defined by analysis of the published pneumococcal genomes;

(b) substrates as from literature and genomic context.

(c) indel insertion/deletion.

(d) in the text and on Figure 1 the operon of Hungary 19 is described, since the one of G54 is not complete.

**Table 2:** Genomic distribution of pneumococcal carbohydrate uptake systems.

species	Strain*	type	ST	SP0062**	SP0092	SP0250	SP0283	SP0310	SP0324	SP0394	SP0474	SP0577	SP0647	SP0758	SP0845	SP0877	SP1185	SP1328	SP1619	SP1683	SP1684	SP1688	SP1722	SP1796	SPH1925	SP1884	SP1897	SP2022	SP2038	SP2108	SP2129	SP2162	SP2184
S.pneumoniae	INV104	1	227	+	+	+	+	+	+	+	+	+	+	+	+	+	a	+	−	+	+	+	+	a	−	+	+	+	+	+	+	+	+
S.pneumoniae	P1031	1	303	+	+	+	+	−	+	−	+	+	+	+	+	+	+	a	+	+	+	+	+	a	fs	+	+	+	+	+	+	+	+
S.pneumoniae	D39/R6	2	595	+	+	+	+	+	+	+	+	+	+	+	+	+	+	a	−	+	+	+	+	a	−	+	+	+	+	+	+	+	fs
S.pneumoniae	SP3 -BS71	3	180	+	+	+	+	+	+	−	−	+	+	+	+	+	+	−	−	fs	+	fs	a	+	+	+	+	+	+	+	fs	+	+
S.pneumoniae	TIGR4	4	205	+	+	+	+	fs	+	+	+	+	+	fs	+	+	+	+	+	+	+	+	+	+	−	+	+	+	+	+	+	+	+
S.pneumoniae	70585	5	289	+	+	+	+	+	+	+	+	+	+	+	+	+	+	a	+	+	+	+	+	+	+	+	+	+	+	+	+	a	+
S.pneumoniae	CDC1873-00	6A	376	+	+	+	+	+	+	+	+	+	+	+	+	fs	+	+	−	+	+	+	+	+	fs	+	+	+	+	+	+	+	+
S.pneumoniae	SP6 -BS73	6A	460	+	+	+	+	fs	+	−	+	+	+	+	fs	+	+	−	−	+	fs	fs	+	+	−	−	+	+	+	+	+	+	+
S.pneumoniae	TIGR6706B	6B	90	+	+	+	+	+	+	+	+	+	+	+	+	fs	+	+	+	+	+	+	+	+	−	+	+	+	+	+	+	a	+
S.pneumoniae	CDC1087-00	7F	191	+	+	+	+	+	fs	−	−	+	+	+	+	+	+	−	+	fs	+	+	+	a	+	+	+	+	+	+	+	a	+
S.pneumoniae	SP9 -BS68	9F		+	+	+	+	−	+	−	+	+	+	+	+	+	a	+	−	+	+	+	+	+	+	+	+	+	+	+	+	+	+
S.pneumoniae	SP195	9V	156	+	+	+	+	−	+	−	+	+	+	+	+	+	a	−	−	+	+	+	+	+	+	+	+	+	+	+	+	+	+
S.pneumoniae	SP11 -BS70	11A	62	+	+	+	+	−	+	−	+	+	+	+	+	+	a	+	−	+	+	+	a	+	+	+	+	+	+	+	+	+	+
S.pneumoniae	AP200	11A	62	+	+	+	+	−	+	−	+	+	+	+	+	+	a	+	−	+	+	+	a	+	+	+	+	+	+	+	+	+	+
S.pneumoniae	MLV-016	11A	62	+	+	+	+	−	+	−	+	+	+	+	+	fs	a	+	−	+	+	+	a	+	+	+	+	+	+	+	+	+	+
S.pneumoniae	CDC288-04	12F	220	+	+	+	+	−	+	−	−	+	+	+	+	fs	+	+	−	+	+	+	+	+	−	+	+	+	+	+	fs	+	+
S.pneumoniae	INV200	14	9	+	+	+	+	−	+	+	−	+	+	+	+	+	a	+	−	+	+	+	+	+	−	+	+	+	+	−	+	+	+
S.pneumoniae	JJA	14	66	+	+	+	+	−	+	+	−	−	+	+	+	fs	a	+	−	+	+	+	+	+	+	+	+	+	+	+	+	+	+
S.pneumoniae	SP14 -BS69	14	124	+	+	+	+	−	fs	−	+	fs	+	fs	fs	+	+	a	−	fs	fs	+	+	+	−	+	+	+	+	+	fs	+	+
S.pneumoniae	SP18 -BS74	18C		+	fs	+	+	+	+	+	fs	+	+	+	+	+	a	a	−	+	+	+	+	+	−	fs	+	+	+	+	+	+	+
S.pneumoniae	19A Hungary 6	19A	268	+	+	+	+	−	+	+	+	+	+	+	+	+	+	+	+	+	−	+	+	a	+	+	+	+	+	+	+	+	+
S.pneumoniae	CDC3059-06	19A	199	+	+	+	+	−	+	−	−	+	+	+	+	+	a	a	−	+	+	+	a	+	fs	+	+	+	+	+	+	+	+
S.pneumoniae	19F Taiwan 14	19F	236	+	+	+	+	+	+	−	+	+	+	+	+	+	+	−	+	+	+	+	+	+	−	+	+	+	+	+	+	+	+
S.pneumoniae	G54	19F	63	+	+	+	+	−	+	+	+	+	+	fs	+	fs	+	+	−	+	+	+	+	a	fs	+	+	+	fs	+	fs	a	+
S.pneumoniae	SP19 -BS75	19F	485	+	+	+	+	+	+	+	−	+	+	+	fs	+	+	−	−	+	+	+	a	+	+	+	+	+	+	+	+	+	+
S.pneumoniae	SP23 -BS72	23F	37	+	+	+	+	+	+	+	+	fs	+	+	+	+	+	−	−	+	+	+	+	a	+	+	+	+	+	+	+	+	+
S.gordonii	V288			+	+	+	+	+	−	−	−	−	+	+	+	+	+	−	+	−	−	+	+	a	−	+	−	−	−	+	−	−	+
S.mitis	B6			+	−	−	+	−	−	−	−	−	+	+	+	+	+	+	+	+	−	−	+	−	−	−	−	−	+	+	+	−	+

* strains are described in reference [32];

** Transport systems are numbered as in TIGR4 according to the EIIC gene in case of PTS and to the substrate binding protein for ABC transporters.

+, presence of the operon; −, absence of the operon; fs, frame shift in a gene of the operon (no re-sequencing of genome data done); a, allelic variant of the transporter with respect to TIGR4.

The availability of genome data allowed to show that these transport systems and the respective operons were components of the variable part of the pneumococcal genome [29]–[32]. It is important to note that bacterial carbohydrate uptake operons are generally functional units and include, in addition to the transporter genes also genes for glycosyl-hydrolases for generation of mono- or disaccharides, enzymes for the metabolic steps linking the specific sugar to glycolysis and usually a regulator (Figure 1). The genomics of the complete complement of prokaryotic transporters and especially carbohydrate uptake systems has been analysed for a number of organisms [21], [33]–[36], but no systematic functional analysis has been carried out. In the present work we describe the functional correlation of carbohydrate related phenotypes and genotypes in a human pathogen with one of the highest number of carbohydrate uptake systems when compared to its genome size [12], [35], [37]. The analysis includes combined phenotypic and genotypic analysis of twenty-six sequenced pneumococcal strains and a systematic functional analysis of a large series of pneumococcal carbohydrate uptake mutants. The aim of this work is to provide a functional genomics annotation of carbohydrate transporters in an important pathogen carrying up to thirty such uptake systems.

## Results

Carbohydrate uptake systems account for a large fraction of the pneumococcal chromosome and include a series of PTS systems, and carbohydrate specific ABC transporters, glycerol permeases and one sodium∶solute symporter. In this work we performed a functional genomics analysis of the pneumococcal carbohydrate uptake systems and related operons, by taking a tripartite approach which includes detailed description of all transport systems, the analysis of their variation at the genomic level in a series of sequenced strains and a functional analysis of knock out mutants for most of the transport systems. The following paragraphs report the genomic context and functional properties of each transporter (Figure 1), starting from the PTS transporters and following on with the ABC transporters, the sodium symporter, the permeases and a brief analysis of related transport systems. The relative genomic and functional characterisation of each transport system is reported in separate sections of the manuscript.

### Carbohydrate uptake loci

#### SP0061-2-3-4 mannose type PTS

This mannose type PTS system is encoded by the genes SP0061 (EIIB, NP_344610.1), SP0062 (EIIC, NP_344611.1), SP0063 (EIID, NP_344612.1), SP0064 (EIID, NP_344613.1); upstream and downstream in the same operon, there are a surface beta-galactosidase (SP0060, *bgaC*), a tagatose-6-phosphate isomerase (SP0065, *agaS*) and an aldose 1-epimerase (SP0066, *galM*). Downstream from this operon, on the opposite strand, a transcriptional regulator of the GntR family (SP0058) is present (Figure 1). Even if not co-expressed, the nearby SP0057 codes for an N-acetyl-glucosaminidase StrH with specificity for GlcNAcβ1-2Man which together with the other glycosyl hydrolases, NanA and BgaA, catalyzes the sequential deglycosylation of human glycoconjugates [38].

#### SP0248-9-50 lactose type PTS

SP0248-9-50 genes code for EIIA (NP_344787.1), EIIB (NP_344788.1) and EIIC (NP_344789.1) subunits of a lactose type PTS. The chromosomal region of this PTS includes upstream two genes coding for two transcriptional regulators, one of the DeoR family and the other with a sugar-binding domain (SP0246 and SP0247), while downstream there are genes for a pyruvate formate lyase SP0251, a formate C-acetyltransferase (SP0252), a fructose-6-phosphate aldolase GldA (SP0253), and a glycerol dehydrogenase [39] (Figure 1). A binding site for the PflR regulator has been mapped upstream of SP0245, SP0246, and SP0251, suggesting that the whole region, including the PTS, is involved in sugar uptake for mixed-acid fermentation [39], [40].

#### SP0282-3-4 mannose type PTS

**Figure 2:**
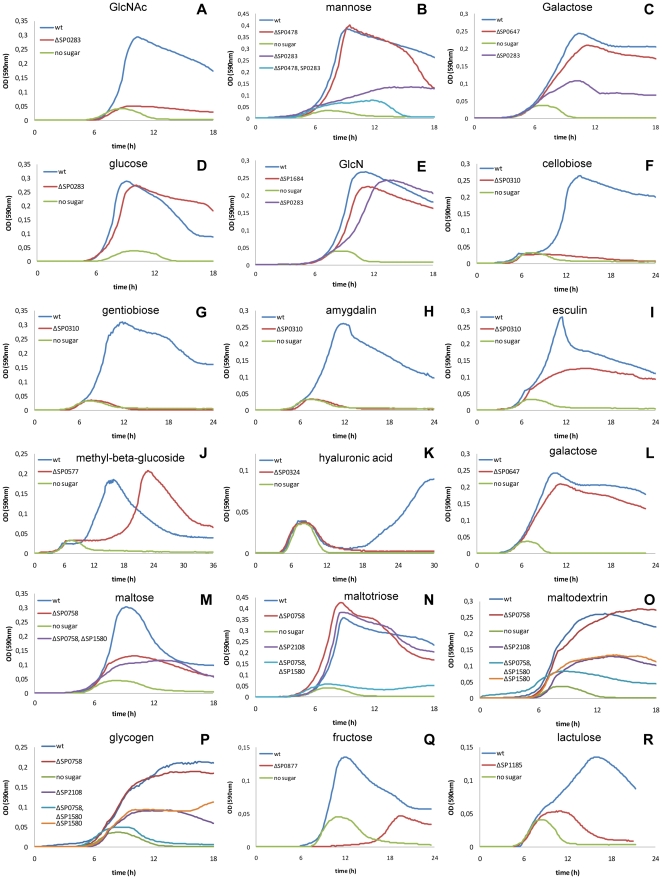
Growth profiles of sugar uptake system mutants on different carbon sources. Pneumococci were grown in CAT medium containing 0.3% of the sugar indicated above every single panel and OD590 nm values were recorded at 10 minute intervals automatically in a thermostatic 96 well microplate reader. In all panels growth of the wild type strain DP1004 on the respective sugar is shown in blue while its growth in CAT without added sugar is shown in green. Growth on N-acetylglucosamine (GlcNAc) is shown in panel A, mannose (panel B), galactose (panel C), glucose (panle D), glucosamine (GlcN) (panel D), the beta-glucosides cellobiose, gentiobiose, amygdalin, esculin, 1-O-methyl-betaglucoside in panel F-J, hyaluronic acid (panel K, galactose (panel L9, maltose (panel M), maltotriose (panel N), maltodextrin (panel O), glycogen (panel P), fructose (panel Q) and lactulose (R). All mutants shown are generated in the D39 derivative DP1004.

The SP0282-3-4 PTS transports glucose and mannose and shows in addition specificity also for galactose, GlcNAc, and GlcN (see below and Fig. 2). This PTS is encoded by SP0282 (*manN*, NP_344820.1) for the subunit IID, SP0283 (*manM*, NP_344821.1) for IIC and SP0284 (*manL*, NP_344822.1) for IIAB (Figure 1) and is conserved in all sequenced genomes. In Firmicutes *manLMN* is usually followed by *manO*, a gene encoding a protein of unknown function. The *manO* gene is lacking in *S. pneumoniae*, the operon contains no additional genes and no regulatory proteins are encoded adjacent to it. The ManLMN PTS had been identified and characterized in *S. salivarius* [41] as a glucose mannose PTS and was shown to be also responsible for uptake of glucose, mannose, fructose and GlcNAc [42]–[44]. In the related species *L. lactis*, glucose uptake is, in addition to the SP0282-3-4 orthologue PtnABCD, also catalyzed by the PtcABC PTS and the GlcU uptake protein, but none of the latter two is present in pneumococcus [44]. Moreover, the orthologous PTS in the Gram positive pathogen *Listeria monocytogenes* (sharing 69% of homology), the species with the highest number of PTS systems, was found to be the main glucose transporter [45], [46].

#### SP0305-8-10 lactose type PTS

The beta-glucoside transporter SP0305-8-10 is a lactose-type PTS composed of three separate subunits: *celB* (EIIB, SP0305, NP_344842.1), *celC* (EIIA, SP0308, NP_344845.1), and *celD* (EIIC, SP0310, NP_344847.1) within an operon containing also a multidomain transcriptional regulator [26], [47]. Upstream in the same locus, pneumococci carry the BglA beta-glucosidase (Figure 1).

#### SP0321-3-4-5 mannose type PTS

SP0321-3-4-5 is a mannose-type PTS for glycosaminoglycan disaccharides and is composed of four separate subunits: EIIA (SP0321, NP_344857.1), EIIB (SP0323, NP_344859.1), EIIC (SP0324, NP_344860.1) and EIID (SP0325, NP_344861.1). The genes coding for the transporter are part of the RegR regulon, which is composed of at least three adjacent transcriptional units [48]. SP0322 codes for a glucoronyl hydrolase which was previously demonstrated to catalyze the release of an unsaturated glucuronic acid from sulphated glycosaminoglycan disaccharides [49]. SP0327 codes for an heparinase like protein and SP0330 (*regR*) codes for the sugar binding transcriptional regulator RegR [48] (Figure 1). On the negative strand, in the opposite direction, another operon, also present in *S. pyogenes*, is part of the same regulon. The four genes of this operon, SP0317 (2-dehydro-3-deoxyphosphogluconate aldolase), SP0318 (2-keto-3-deoxygluconate kinase), SP0319 (hexose-6-phosphate isomerase), SP0320 (5-keto-D-gluconate 5-reductase) (Figure 1) are involved in the utilization of ketogluconate [50]. Upstream of this operon there is the gene for the extracellular hyaluronate lyase (SP0314), an important pneumococcal virulence factor that catalyzes the release of disaccharides from glycosaminoglycans of the extracellular matrix of soft connective tissues [51]. These glycosaminoglycan disaccharides are the appropriate substrates for the intracellular SP0322 hydrolase and the possible substrates for the SP0321-3-4-5 PTS [49].

#### SP0394-6 galactitol type PTS

SP0394-6 PTS is a galactitol-type PTS which transports mannitol and is composed of an EIIBC (*mtlA*, SP0394, NP_344918.1) and an EIIA subunit (*mtlF*, SP0396, NP_344920.1) [52]. The highly conserved orthologue operon in other Firmicutes has been demonstrated to be regulated by the PRD-containing transcriptional activator MtlR [53], and mannitol metabolism to be dependent on the downstream mannitol-1-phosphate 5-dehydrogenase *mtlD* (SP0397) (Figure 1) [52]. The orthologous operon has been well described in *Streptococcus mutans* [54].

#### SP0476-8 lactose type

The SP0476-8 operon encodes an EIIA domain (SP0476, *lacF-1*, NP_344995.1), an intracellular 6-phospho-beta-galactosidase (SP0477, *lacG-1*) and an EIIBC domain (SP0478, *lacE-1*, NP_344997.1). Upstream from this operon there is a regulon which contains the genes for a lactose type EIIC PTS subunit (SP0474, NP_344993.1), an hypothetical protein (SP0475) and, on the opposite strand, a transcriptional regulator of the ROK family (SP0473) (Figure 1).

#### SP0577 glucose type PTS

The SP0577 (NP_345091.1) gene encodes a BglP glucose-type PTS composed of an EIIA, EIIB, and EIIC domain, present in a three-gene operon with the transcription antiterminator (*licT*, SP0576) upstream and the 6-phospho-beta-glucosidase (*bglA-2*, SP0578) downstream (Figure 1). The operon, including the LicT regulator and the BglA beta-glucosidase, has been characterized in many species including *S. mutans*, where the BglP transporter is responsible for uptake of the beta-glucoside aesculin in the presence of glucose [55].

#### SP0645-6-7 galactitol type PTS

SP0645-6-7 PTS is a galactitol-type PTS which transports galactose, and was reported to be induced also by lactose and galactitol [56]. SP0645-6-7 is composed of an EIIA (*gatA*, SP0645, NP_345152.1), EIIB (*gatB*, SP0646, NP_345153.1), EIIC (*gatC*, SP0647, NP_345154.1) subunit. The transporter is, despite a long intergenic region, co-transcribed with the downstream *bgaA* gene encoding a surface exposed beta-galactosidase [56], [57] (Figure 1).

#### SP0758 glucose type PTS

The maltose transporting SP0758 glucose-type PTS is composed of an EIIA, EIIB, and EIIC domain (MalT, NP_345256.1) (Figure 1). SP0758 in many genome sequences is followed by a small ORF, which is a mis-annotation in TIGR4 and covers the appropriate start site of SP0760 (appropriately translated in R6; spr0669). In *S. pyogenes* the operon was found to be competitively regulated by the MalR regulator and CcpA [58]. SP0758 MalT orthologues in *E. faecalis*, *S. mutans* and *S. pyogenes* have also been described [58]–[60]. *B. subtilis* also possesses a PTS transporter for maltose encoded by the *malP* gene [61] . This gene is organized in an operon together with the *malA* gene, which codes for a 6-P-α-glucosidase, which splits maltose-6-P into glucose-6-P and glucose [62]. Nevertheless, as will be mentioned below (see SP1884), trehalose-6-P was reported to be split by trehalose-6-P phosphorylase into glucose-1-P and glucose-6-P. This is possible because trehalose is a symmetric molecule containing two non-reducing glucose moieties connected by an α,α-1,1-glycosidic bond [74]. In contrast, the detailed metabolism of maltose after its uptake by the PTS of the four above bacteria remains obscure. Interestingly, in all four organisms *malT* seems to be organized in an operon together with a gene encoding a protein of unknown function (EF0960 in *E. faecalis* and SP0760 in *S. pneumoniae*). It is likely that this protein plays an important but yet unknown role in maltose transport.

#### SP0877 fructose type PTS

The SP0877 (NP_345364.1) fructose transporter is the highly conserved fructose-type PTS of 650 amino acids composed of EIIA, EIIB, and EIIC domains and is responsible for uptake of fructose [63] (Figure 1). The genetic organization and regulation of the fructose operon has recently been analysed in the model microorganism *L. lactis* [64]. SP0877 (*fruA*), as in other species, is co-transcribed with the upstream *fruR* repressor and the *fruC* 1-phosphofructokinase. The whole operon is conserved in all pneumococcal genome sequences and in related species. A FruR binding site and a *cre* site could be found upstream from SP0875, but the latter is less evident than in *L. lactis* [64].

#### SP1185 lactose type PTS

The SP1185-6 lactulose and lactose transporter is composed of EIIBC (NP_345654.1) and EIIA (NP_345655.1) subunits of the lactose type. In pneumococci this regulon also includes genes coding for enzymes of the tagatose pathway, such as (from upstream) *lacX* (SP1194) (truncated in all pneumococcal strains), galactose-6-phosphate isomerase subunit A (SP1193, *lacA*) and subunit B (SP1192, *lacB*), tagatose-6-phosphate kinase (SP1191, *lacC*), tagatose 1,6-diphosphate aldolase (SP1190, *lacD*), 6-phospho-beta-galactosidase (SP1184, *lacG-2*). Furthermore, within this operon, a transcription antiterminator (SP1187, *LacT*) and, on the opposite strand, the lactose phosphotransferase system repressor (SP1182, *lacR-2*) are present (Figure 1). This operon is essentially identical in *S. mutans* and *L. lactis*, except for LacR regulator and LacX [65]–[68]. The tagatose 6-phosphate operon is not present in the other model organisms *S. salivarius* and *S. thermophilus*. In *S. mutans* the orthologue PTS (smu.1492) has been shown to be over-expressed in the presence of lactose and galactose [69].

#### SP1617-8-9 fructose type PTS

The SP1617-8-9 PTS is a fructose-type PTS composed of EIIA (SP1619, NP_346060.1), EIIB (SP1618, NP_346059.1), and EIIC (SP1617, NP_346058.1) domains. This transporter is part of an operon with an upstream multi-domain transcriptional regulator (SP1621) containing two helix-turn-helix domains, two PTS regulatory domains and one EIIB domain. Downstream an EIIA domain gene is located, annotated as a nitrogen regulatory component. Although the two genes are separated in all strains, it is tempting to suggest that this EIIA protein interacts with both the multidomain transcriptional regulator and the transporter. Downstream, within the same operon, two genes coding for enzymes of the pentose phosphate pathway are found: SP1616 coding for a ribulose-phosphate 3-epimerase [70] and SP1615 (*recP-1*) for a transketolase [71] (Figure 1).

#### SP1684 glucose type PTS

All pneumococcal strains carry a regulon for sialic acid metabolism, which contains three transporters and is composed of four transcriptional units [72]. The third of these operons encodes the Glc-PTS SP1684 (NP_346123.1) for glucosamine (GlcN) (composed of only the EIIB and EIIC domains), the NanE epimerase SP1685, an ABC transporter (see below) and a sialic acid lyase and kinase. This transcriptional unit, as well as the others containing *nanA* and *nanB* appears to be regulated by the phospho-sugar binding transcriptional regulator SP1674 located on the reverse strand, whose deletion abolishes transcription of the operon (unpublished data).

#### SP1722 glucose type PTS

SP1722 is a gene encoding an EIIBCA domain of the glucose-type PTS for sucrose (*scrH*, NP_346159.1), co-transcribed with the *scrK* fructokinase (SP1721) [27]. On the opposite strand, there are a sucrose-6-phosphate hydrolase (SP1724, *scrB*) and a *scrR* sucrose operon repressor (SP1725) which has its binding sites 108 bp upstream from the SP1722 gene and 105 bp upstream from the SP1724 gene.

#### SP1884 glucose type PTS

The SP1884 gene encodes a glucose type PTS for trehalose of 655 amino acids composed of EIIA, EIIB, and EIIC domains (NP_346277.1). The three genes SP1883 (dextran glucosidase DexS), SP1884 (PTS^TRE^), and SP1885 (gntR) appear to be part of a functional unit, since all three are deleted in strain SP6-BS73. The SP1884 PTS is part of a very conserved family sharing 50% identity with the first trehalose PTS described in *Vibrio parahaemolyticus* [73]. In contrast to *L. lactis* MG1363, where the genes for the PTS are organized in an operon together with the genes for a trehalose-6-P phosphorylase (TrePP) and a phosphoglucomutase [74], no genes encoding catabolic enzymes are associated to the trehalose-specific PTS genes in *S. pneumoniae*, including *trePP*. It is therefore likely that trehalose-6-P is split by SP1885, which probably functions as a 6-P-α-glucosidase. Upstream on the opposite strand, a GntR type regulator (SP1885) is found (Figure 1).

#### SP2022-3-4 lactose type PTS

SP2022-3-4 is a lactose type PTS composed of an EIIC (SP2022, NP_346447.1), EIIB (SP2023, NP_346448.1) and an EIIA domain (SP2024, NP_346449.1). Upstream of the genes coding for the PTS, a beta-glucosidase is present sharing 54% identity with the mannoside-phospho-beta-glucosidase *gmuD* of *B. subtilis*, a hydrolase found in an operon responsible for the metabolism of glucomannans (branched sugars of beta-linked mannoses and glucoses). Upstream, on the negative strand, a transcriptional regulator of the GntR family is found.

#### SP2036-7-8 ascorbate type PTS

The SP2036-7-8 genes encode EIIA (NP_346461.1), EIIB (NP_346462.1) and EIIC (NP_346463.1) components of the asc-type PTS system for ascorbate transport, and are found inside of a nine-genes operon coding for enzymes for ascorbate utilization (SP2035 3-keto-L-gulonate 6-phosphate decarboxylase *ulaD*, SP2034 L-ribulose-5-phosphate 3-epimerase *ulaE*, SP2033 L-ribulose 5-phosphate4-epimerase *ulaF*, SP2031 L-ascorbate 6-phosphate lactonase *ulaG*, SP2030 transketolase *tkt*) and for a multidomain transcriptional regulator (Figure 1), containing two helix-turn-helix domains, two PTS regulatory domains and one EIIB domain. This operon is highly conserved with high homology in other streptococci, such as *S. pyogenes* and *S. mutans*, and the same operon structure could be found even in *E. coli* [75], [76].

#### SP2129-30 ascorbate type PTS

SP2129-30 PTS is an asc-type PTS composed of EIIB (SP2130, NP_346548.1) and EIIC (SP2129, NP_346547.1) subunits that are co-transcribed upstream with a transcriptional regulator (SP2131). This multidomain regulator carries two helix-turn-helix domains, a PTS regulatory domain (PRD), a lactose type EIIB domain and galactiol EIIA domain. Inasmuch the PTS is devoid of a dedicated EIIA protein, it is tempting to suggest that the SP2129-30 PTS is energised by the EIIA domain of the regulator. Downstream genes for the N-terminal and C-terminal domain of a transketolase are found (SP2128 and SP2127) (Figure 1).

#### SP2161-2-3-4 mannose type PTS and SPG2105 CUT-1 ABC transporter

The SP2161-2-3-4 genes code respectively for EIID (NP_346575.1), EIIC (NP_346576.1), EIIB (NP_346577.1) and EIIA (NP_346578.1) components of the mannose type PTS system for fucose, found inside of an eleven-genes regulon composed of the SP2168 fucose operon repressor on the positive strand and, on the negative strand, SP2167 L-fuculokinase (*fucK*), SP2166 L-fuculose phosphate aldolase (*fucA*), SP2165 fucose operon protein FucU, SP2161-2-3-4 PTS system, SP2160 conserved hypothetical protein, SP2159 fucolectin-related protein and SP2158 L-fucose isomerase (*fucI*) (Figure 1). The corresponding promoter has previously been characterized as being induced by fucose and repressed by glucose and sucrose [77]. Fucose is a galactose derivative lacking the 6-hydroxyl group usually phosphorylated by the PTS. The question therefore arises whether the PTS indeed phosphorylates fucose or whether it is transported without phosphorylation, converted inside the cell into fuculose by L-fucose isomerase and subsequently phosphorylated to fuculose-1-P by the fuculokinase. Alternatively, this PTS might transport a fucose-containing oligosaccharide, from which fucose would be released by a hydrolase. Most of the pneumococci analyzed, including D39 and TIGR4, share this type of regulon (Table 2). A different allelic form of this operon is found in the remaining pneumococcal strains, including G54 [14]. The L-fucose isomerase (frame-shifted in G54), L-fuculose phosphate aldolase, L-fuculose kinase, fucose operon repressor and the intergenic region with the promoter are completely conserved in both allelic versions. The striking difference is in the transport region where the PTS is substituted by an ABC transporter (SPG2105-6-7) of the CUT1 family, which transports fucose without phosphorylation. Interestingly, like all the other pneumococcal CUT1 ABC transporters, also this one lacks the ATP binding protein. The substrate binding specificity of this transporter was defined as being the terminal trisaccharides of the A and B blood groups [78]. Furthermore, within this second operon, five new genes are found: SPG2100 and SPG2101 blood group cleaving endo-beta-galactosidases, SPG2102 glycosyl hydrolase, SPG2103 hypothetical protein and SPG2104 alpha-L-fucosidase.

#### SPH1925-6-7 and SPH1929 lactose type PTS

An additional PTS transporter, absent in D39 and TIGR4, is found in about a third of pneumococcal genomes [29], including Hungary 19A-6, to which the following gene numbering refers. SPH1925 and SPH1929 are two genes coding for two EIIC (YP_001695180.1, YP_001695184.1) lactose type PTS subunits, found in the same operon. Adjacent to SPH1925 there are the genes for EIIA (SPH1926, YP_001695181.1) and EIIB (SPH1927, YP_001695182.1), which are separated from the SPH1929 EIIC by a phospho-3-sulfolactate synthase of the ComA family (SPH1928). Downstream of SPH1925, genes for a sulfatase (SPH1924), a sulfatase modifying enzyme (SPH1923) and a sulfite exporter of the TauE family (SPH1922) are present (Figure 1). The operon is most probably linked to the transcriptional regulator SP1930 encoded on the opposite strand which is composed of a MarR DNA binding and ROK sugar binding domain. EIIC SPH1925 and SPH1929 seem to have very few orthologues across eubacteria.

#### SP1176-7 PTS general components

PTS transporters are multi-component transporters with a sugar specific EII complex and two general enzymes, HPr (phosphocarrier protein) and EI (enzyme I) (Figure 1). *PtsH* (SP1177, HPr, NP_345646.1) and *ptsI* (SP1176, EI, NP_345645.1) are co-transcribed and the locus is highly conserved as in all related species [18].

#### SP0090-1-2 CUT1 ABC transporter

CUT1-family ABC transporter SP0090-1-2 is found in an operon, conserved all over the sequenced genomes of *S. pneumoniae*, with three ORFs which code for the permeases (SP0090 and SP0091, NP_344637.1 and NP_344638.1) and the substrate-binding lipoprotein (SP0092, NP_344639.1) (Figure 1).

#### SP0845-6-7-8 CUT2 ABC transporter

SP0845-6-7-8 is the only member of the second family of the carbohydrate uptake transporter of the ABC transporter superfamily (CUT2) [22]. Unlike the members of the pneumococcal CUT1 family, the genes coding for this transporter also include the ATPase subunit with two ATP-binding domains (SP0846, NP_345337.1), adjacent to the substrate-binding protein (SP0845, NP_345336.1) and the two permeases (SP0847 and SP0848, NP_345338.1 and NP_345339.1). These genes are situated in an operon which also codes for putative ribonucleoside metabolic enzymes: *cdd* (SP0844, cytidine deiminase), *deoC* (SP0843, deoxyribose-phosphate aldolase), *pdp* (SP0842, pyrimidine-nucleoside phosphorylase) and an hypothetical protein (SP0841) with a methyl transferase domain [79] (Figure 1). CUT2 transporters in most species are composed of a homodimer permease, while in *S. mutans* and *S. pneumoniae* the locus encodes two permeases, predicted to function as heterodimers [22], [79].

#### SP1681-2-3 and SP1688-9-90 CUT1 ABC transporter

Three transporters are part of the sialic acid regulon and include the SP1681-2-3 and SP1688-9-90 CUT1 ABC transporter in addition to the SP1684 PTS for glucosamine [72]. This operon is intimately connected to the virulence of pneumococci, including biofilm formation, adhesion, colonization, pneumonia and sepsis [9], [72], [80], [81] and its importance for the metabolic use of sialic acid in complex host carbohydrates was described [38]. The upstream operon encodes the surface located pneumococcal neuraminidase NanA SP1693, and the second operon a hypothetical protein, a CUT1 ABC transporter composed of a substrate binding lipoprotein SP1690 (NP_346129.1) and two permeases (SP1688-9, NP_346127.1 and NP_346128.1; ManNAc and NeuNAc transport), the neuraminidase NanB (SP1687) and a sialic acid mutorotase. The third operon encodes the NanE epimerase SP1685, a Glc-PTS SP1684 for Glc (see above), followed by another CUT1 ABC transporter composed of a substrate binding lipoprotein (SP1683, NP_346122.1) and two permeases (SP1681-2, NP_346120.1 and NP_346121.1; ManNAc and NeuNAc transport) and a sialic acid lyase and kinase [28].

#### SP1796-7-8 CUT1 ABC transporter

The CUT1-family ABC transporter SP1796-7-8 for sucrose, composed of a substrate binding protein (SP1796, *susX*, NP_346229.1) and two permeases (SP1797, *susT-1*, NP_346230.1, and SP1798, *susT-2*, NP_346231.1), was firstly identified by Iyer and Camilli [27] for the presence of a sucrose-binding box indicated by a signature beta-fructosidase motif (NDPNG) [82] which is conserved across bacteria, planta and fungi, likewise its paralogue PTS SP1722. This ABC transporter is found in an operon with a sucrose-6-phosphate hydrolase (SP1795, *susH*) and a regulator of the LacI family (SP1799, *susR*) (Figure 1).

#### SP1895-6-7 CUT1 ABC transporter

SP1895-6-7 is a CUT1-family ABC transporter (*rafGFE*) for α-galactosides, where the SP1895 (NP_346326.1) and SP1896 (NP_346327.1) genes code for two permeases and SP1897 (NP_346328.1) for a substrate-binding protein. As for the other pneumococcal CUT1 ABC transporters no protein with an ATP binding cassette is encoded in the operon. The transporter is reported to be co-transcribed with the downstream SP1894 sucrose phosphorylase (*gtfA*) and the uncharacterized gene *rafX*. Downstream, the SP1898 α-galactosidase (*aga*) is present with a *cre* site at −39 bases. The *aga* expression is controlled by two genes, the activator *rafR* (SP1899), an AraC-type DNA-binding domain-containing protein, and the repressor *rafS* (SP1900) (Figure 1), which is reported being induced in the presence of raffinose and repressed in the presence of sucrose [25].

#### SP2108-9-10 CUT1 ABC transporter

The SP2108-8-10 ABC transporter for malto-oligosaccharides is composed of three genes: the SP2108 substrate-binding (*malX*, NP_346527.1) and the SP2109 (*malC*, NP_346528.1) and SP2110 (*malD*, NP_346529.1) permeases. In the same regulon, 4 other genes are present: the SP2111 maltodextrose utilization protein's gene (*malA*), SP2112 HTH type transcriptional regulator (*malR*) [24] , and upstream on the opposite strand, the SP2107 4-alpha-glucanotransferase (*malQ*) and the SP2106 maltodextrin phosphorylase (*malP*) [83] (Figure 1). MalX has been recently described to have high affinity for malto-oligosaccharides ranging from three to eight residues and low affinity for the disaccharide maltose or for molecules with more than eight residues [84].

#### SP1580 CUT1 ATP-binding cassette domain protein

SP1580 encodes a 376 amino acid protein (MsmK, NP_346026.1) (Figure 1) with one ATP-binding cassette domain and a C-terminal TOBE (transport associated) domain, predicted to function as a homodimer [22]. SP1580 is the only ATP-binding cassette domain of the CUT1 family in *S. pneumoniae* [23]. Its relationship with the six permease heterodimers and substrate binding proteins of the CUT1 family is discussed below.

#### SP1328 sodium-solute symporter

The SP1328 transporter (NP_345786.1) is involved in sialic acid transport and is, in distinction to all other pneumococcal sugar transporters, a solute∶sodium symporter (SSS family; TC2.A.21). In the transporter database (http://www.tcdb.org/), the protein with the highest similarity is the sodium-sialic acid co-transporter *nanT* of *Vibrio fischeri* and *E. coli* [85], [86]. This secondary transporter has the usual 12 membrane-spanning domains found in most members of the Major Facilitator Superfamily. Members of the SSS family catalyze solute uptake via Na^+^ symport. The whole locus containing SP1328 is well defined by the conserved sequences which become evident by comparison of pneumococci not carrying this locus. The 21 kb genomic fragment of this locus contains genes for proteins involved in sialic acid uptake and metabolism, and downstream the series of genes *nptA-K* encoding the subunits of a V type sodium ATPase [87]. As this ATP dependent sodium exporter is present only in strains harbouring a sodium∶solute co-importer in the same locus, the two transporters may constitute a functional unit. Surrounding the SP1328 symporter are found the necessary enzymes for sialic acid utilization including the neuraminidase NanC, a kinase, a lyase, an epimerase, an oxidoreductase and a phosphosugar binding transcriptional regulator [88] (Figure 1). In *C. pefringens* and *S. aureus*, which are both positive for sialic acid metabolism, this sodium solute symporter represents the only sialic acid transporter [86].

Pneumococci not carrying the above locus may either carry an alternative 13.5 kb locus or no intervening sequences between the conserved genome backbone. The alternative locus carries genes encoding a putative oligopeptide ABC transporter (spr1191-4) composed of a substrate binding protein, two permeases and an ATP binding cassette protein and two additional genes still linked to the amino sugar metabolism which are a sialic acid mutorotase and an amino-sugar epimerase. This type of locus appears to be rearranged, since there are differences amongst strains and some genes appear to be frame shifted.

#### SP2184 glycerol facilitator

SP2184 (NP_346595.1) is a typical glycerol facilitator belonging to the MIP family of transporters (TC1.A.8). The *glpF* facilitator is part of a highly conserved operon also including the upstream *glpD* glycerol-3-phosphate dehydrogenase (SP2185) and the *glpK* glycerolkinase (SP2186) (Figure 1) [89], [90].

#### SP1491 glycerol facilitator (putative)

SP1491 (NP_345943.1) is, in addition to SP2184, the second pneumococcal MIP family transporter (TC1.A.8) annotated as a putative glycerol facilitator.

### Other possible carbohydrate related transporters

It is difficult to draw a precise border between transporters involved in carbohydrate and carbon-source uptake and other transporters. While this is easy for the specialized group of PTS transporters this is less easy for the ABC transporters and even less for symporters and facilitators. Examples include (1) the amino acid type ABC transporter spr1192 for amino sugars, the allelic variant of the amino sugar sodium symporter SP1328, described in this work (Table 1 and below), (2) the MIP family proteins annotated as putative glycerol facilitator SP1491 and the aquaporin SP1778 (NP_346211.1), not discussed in this work, (3) the MSF (major facilitator superfamily) SP_1587 (NP_346033.1) oxalate∶formate antiporter (TC 2.A.1), and (4) the three APC transporters (Amino acid-Polyamine-organocation superfamily; TC 2.A.40) SP0287 (NP_344825.1) xanthine/uracil/ascorbate permease, SP1848 (NP_346280.1) xanthine permease and SP1286 (NP_345750.1) uracil permease. From the phenotypic point of view similar problems arise; i.e. pneumococci are sensitive to fosfomycin, which is usually taken up by the hexosephosphate transporter UhpT or the glycerol-3-phosphate transporter GlpT [91]. However, no homologue of either one of these transporters is present in pneumococci [92].

### Genomic and metabolic variability

Comparison of the three reference strains showed minor differences in carbohydrate utilisation, including variability of N-acetyl-neuraminic acid (sialic acid), beta-glucosides and glycerol utilisation (Table 3). All three differences could be traced to confirmed frame shifts in related metabolic or regulatory genes. The inability of D39 to metabolise sialic acid and glycerol may be correlated to a frame shift in its N-acetyl-neuramate lyase gene within the *nanA-nanB* operon for sialic acid [38] and in the *glpD* glycerol-3-phosphate dehydrogenase gene, respectively. The inability of TIGR4 and G54 to ferment beta-glucosides, except for salicin, could be explained by a frame shift in the regulator of the beta-glucoside operon in TIGR4 and the complete absence of this operon in G54 [26].

**Table 3:** Carbohydrate fermentation profile of *S. pneumoniae* strains.

Species	Strain*	type	ST	fructose**	galactose	glucose	mannose	GlcNAc	maltose	sucrose	tr ehalose	amygdalin	arbutin	cellobiose	gentiobiose	salicin	melibiose	raffinose	stachyose	lactose	glycogen	inulin
*S.pneumoniae*	INV104	1	227	5	5	5	4	1	4	4	2	3	1	5	5	4	1	1	5	4	1	1
*S.pneumoniae*	P1031	1	303	5	5	5	5	5	5	5	5	1	1	1	1	1	3	5	5	5	3	2
*S.pneumoniae*	D39	2	595	5	5	5	5	5	5	5	5	5	5	5	5	5	5	5	5	5	5	1
*S.pneumoniae*	SP3 -BS71	3	180	5	2	4	2	2	5	5	3	1	2	2	5	1	1	2	1	5	1	1
*S.pneumoniae*	TIGR4	4	205	5	5	5	5	5	5	5	5	1	1	1	1	1	5	5	5	5	1	2
*S.pneumoniae*	70585	5	289	5	5	5	5	5	5	5	5	4	2	5	5	5	3	5	5	5	4	2
*S.pneumoniae*	CDC1873-00	6A	376	5	5	5	5	3	5	5	5	5	1	5	5	5	1	5	2	5	2	1
*S.pneumoniae*	SP6 -BS73	6A	460	5	5	5	5	5	4	5	1	5	1	5	5	5	2	3	2	4	3	2
*S.pneumoniae*	TIGR6706B	6B	90	4	4	5	5	1	5	5	3	3	3	5	5	5	1	3	5	4	2	2
*S.pneumoniae*	CDC1087-00	7F	191	5	5	5	5	3	5	5	5	1	1	1	1	1	2	5	5	5	1	1
*S.pneumoniae*	SP9 -BS68	9F		5	5	5	5	2	5	5	5	1	1	1	1	1	2	5	5	5	3	3
*S.pneumoniae*	SP195	9V	156	5	5	5	5	1	5	5	5	1	1	1	1	5	2	5	5	5	4	1
*S.pneumoniae*	SP11 -BS70	11A	62	5	3	5	3	1	5	5	5	1	1	1	1	5	1	3	1	5	2	1
*S.pneumoniae*	AP200	11A	62	5	5	5	5	2	5	5	5	1	1	1	1	5	1	5	4	5	3	1
*S.pneumoniae*	MLV-016	11A	62	5	5	5	5	1	5	5	5	1	1	1	1	5	1	5	5	5	1	1
*S.pneumoniae*	CDC288-04	12F	220	5	5	5	5	5	5	5	5	1	1	1	1	5	1	5	5	5	4	4
*S.pneumoniae*	INV200	14	9	5	5	5	5	5	5	5	5	1	1	1	1	4	4	5	5	5	2	2
*S.pneumoniae*	JJA	14	66	5	5	5	5	5	5	5	5	1	1	1	1	1	1	5	3	5	4	2
*S.pneumoniae*	SP14 -BS69	14	124	5	5	5	5	3	5	5	5	1	1	1	1	1	2	5	5	5	4	4
*S.pneumoniae*	SP18 -BS74	18C		5	5	5	5	4	5	5	5	5	1	5	5	5	3	3	5	5	4	3
*S.pneumoniae*	19A Hungary 6	19A	268	5	5	5	5	5	5	5	5	1	1	1	1	5	2	5	5	5	4	1
*S.pneumoniae*	CDC3059-06	19A	199	5	5	5	5	2	5	5	5	1	1	1	1	1	1	5	5	5	4	2
*S.pneumoniae*	19F Taiwan 14	19F	236	5	5	5	5	5	5	5	5	1	1	1	1	1	3	5	5	5	2	2
*S.pneumoniae*	G54	19F	63	5	5	5	5	5	5	5	5	1	1	1	1	5	1	5	5	5	1	1
*S.pneumoniae*	SP19 -BS75	19F	485	5	5	5	5	4	5	5	5	5	1	5	5	5	3	5	5	5	4	4
*S.pneumoniae*	SP23 -BS72	23F	37	5	3	5	4	3	5	5	5	1	1	1	1	1	3	5	4	5	1	1
*S.gordonii*	V288			5	5	5	5	5	5	5	5	5	5	5	5	5	1	1	1	5	5	5
*S.mitis*	NCTC12261			5	5	5	5	5	5	5	1	1	1	1	1	1	3	5	5	5	3	1

* strains are described in reference [32];

** Carbohydrates are grouped according to the structure in monosaccharides, alpha-glucosides, beta-glucosides, alpha-galactosides, beta-galactoside, polysaccharides. Intensity of fermentation, detected as the degree of colour change of a pH indicator, is given in arbitrary units from 1 (no fermentation) to 5 (excellent fermentation).

In order to perform a genome wide correlation of carbohydrate related phenotypes and genotypes we determined the fermentation pattern of twenty carbohydrates for twenty-six sequenced pneumococcal strains (Table 3) [32]. Out of the panel of sugars selected, some were fermented by all strains (fructose, galactose, glucose, mannose, maltose, sucrose and lactose) and some by a number of strains (N-acetyl-glucosamine, trehalose, alpha-galactosides, and beta-glucosides). These metabolic data were compared to the presence of the thirty pneumococcal carbohydrate transporters in the genomes of the strains used in fermentation (Tables 2 and 3) [12], whose genome sequences were available and which were kindly provided for experimental work (Table 2) [32]. In this analysis we did not consider frame shifted genes, since re-sequencing would be needed to differentiate between sequencing errors and true frame shifts. Out of the thirty sugar transporters, sixteen were conserved in all strains. Of the conserved transporters fourteen are PTS transporters, five ABC transporters, the sodium∶solute symporter and the glycerol permease.

Within the twenty-six genomes analyzed, five loci presented an allelic variation of the respective uptake system (Table 2). Upon these are both the sucrose transporters SP1722 PTS and SP1796-7-8 CUT1 ABC. The former presents an allelic variant also belonging to the PTS-Glc family in 30% of the strains analyzed (Table 2), with only 49% overall amino acid identity. An example for the SP1722 allele is YP_003877313 of strain AP200 [93]. Interestingly, the variability is confined to the EIIB and EIIC domains with over 99% identity for the C-terminal EIIA domain. In the case of the SP1796-7-8 ABC transporter operon, seven out of twenty strains presented an allelic form of the locus. An example is the locus in D39 and R6 (spr1618-19-20; NP_359210-11-12), where the locus differs for the ABC transporter subunits (22 to 29% amino acid identity), part of the regulator, but sharing an identical hydrolase with TIGR4. The EIIBC subunit of SP1185 PTS has an allelic variant, also of the PTS-Lac family, with 81% amino acid identity in ten strains analysed. An example is SPAP_1214-5 (YP_003876805.1) of AP200 strain. The *nanC* regulon with the SP1328 sodium-solute symporter is present in thirteen of the analysed genomes while the operon is missing in six strains and a different operon is present in seven strains. An example for the latter locus are D39 and R6, which carry an alternative 13.5 kb locus encoding a putative oligopeptide ABC transporter spr1191-2-3-4, composed of a substrate binding protein (NP_358787.1), two permeases (NP_358785.1 and NP_358786.1) and an ATP binding cassette protein (NP_358784.1) and two additional genes still linked to the amino sugar metabolism which are a sialic acid mutorotase and an amino-sugar epimerase. This type of locus appears to be rearranged, since there are differences amongst strains and some genes appear to be frame shifted. Twenty two out of the twenty six strains, including D39 and TIGR4, share a regulon for fucose utilization containing a SP2161-2-3-4 mannose type PTS as described above. A different allelic form of this operon is found in the remaining pneumococcal strains, including G54 [14]. The main difference in the locus is confined to the transport region and presence of five new genes including SPG2100 and SPG2101, two blood group cleaving endo-beta-galactosidases, SPG2102 glycosyl hydrolase, SPG2103 hypothetical protein and SPG2104 α-L-fucosidase. Regarding the transporter the PTS is substituted by an ABC transporter (SPG2105-6-7) of the CUT1 family (YP_002038755.1, YP_002038756.1, YP_002038757.1) [78]. Interestingly, like all the other pneumococcal CUT1 ABC transporters, also this one lacks the ATP binding protein.

**Figure 3:**
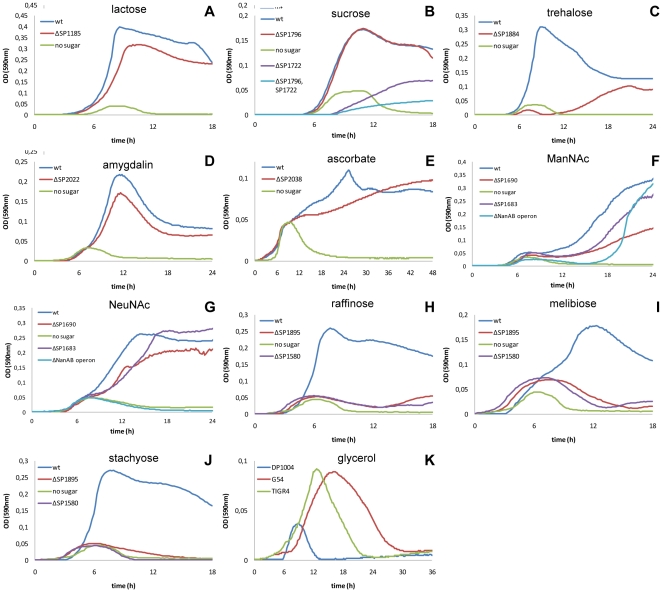
Growth profiles of sugar uptake system mutants on different carbon sources (continued from **Fig. 2**). As for Figure 2 pneumococci were grown in CAT medium containing 0.3% of the sugar indicated above every single panel and OD590 nm values were recorded at 10 minute intervals automatically in a thermostatic 96 well microplate reader. In all panels growth of the wild type strain on the respective sugar is shown in blue while its growth in CAT without added sugar is shown in green. Growth on lactose is shown in panel A, on growth sucrose (panel B), trehalose (panel C), amygdalin (panel D), ascorbate (panel E), N-acetylmannosamine (ManNAc) (panel F), sialic acid (NeuNAc) (panel G), the alpha-galactosides raffinose, melibiose and stachyose (panes H–J) and glycerol (panel K). All mutants shown are generated in the D39 derivative DP1004, except for panels F and G where strain G54 and mutants in G54 were used for evaluation of growth in ManNAc and NeuNAc.

Among the thirty uptake loci, ten were completely absent in at least one of the genomes analysed. While in most cases no defined phenotype could be detected, the observed variability helped in defining the functional unit of the genes of a locus. Phenotype-genotype correlation was found for the beta-glucosides, where nine strains able to ferment gentiobiose and cellobiose carried the intact SP0303-10 operon, and ten strains that lacked the operon were unable to ferment these sugars (Table 3). These data are in accordance with previous work on the identification and characterisation of the beta-glucoside operon in R6 [26]. No genetic evidence, including SNP analysis in the promoter regions, was found for the lack of beta-glucoside fermentation in three strains carrying an apparently intact operon (Tables 2 and 3). Strain SP6-BS73, which was the only strain not fermenting trehalose, was also the only one lacking the SP1884 PTS system (Tables 2 and 3). This genomic correlation was confirmed by generating a mutant (Figure 3E), which definitively confirmed the identification of SP1884 as the trehalose PTS [73].

No genomic correlation could be found to the variability of fermentation phenotypes for the beta-glucoside salicin, N-acetyl-glucosamine (GlcNAc) and the alpha-galactosides. Lack of correlation of certain phenotypes indicates that variation in expression may be at the basis of some of the phenotypic differences.

### Pneumococcal carbohydrate utilisation

When trying to correlate genotypes and phenotypes in *S. pneumoniae*, one of the most important steps was the determination of the global carbohydrate substrate utilisation profile of *S. pneumoniae* by measuring acid generation (fermentation), growth on single carbon sources and metabolic activity using phenotype microarray. Altogether, pneumococci were found to metabolise thirty-two carbohydrates including the three-carbon molecule glycerol, nine hexoses or hexose derivatives (ascorbate, fructose, galactose, glucosamine, glucose, mannose, N-acetyl-glucosamine, N-acetyl-mannosamine, and N-acetyl-neuraminic acid), three alpha-galactosides (melibiose, raffinose and stachyose), two beta-galactosides (lactose, and lactulose), four alpha-glucosides (maltose, maltotriose, sucrose and trehalose), seven beta-glucosides (amygdalin, arbutin, 1-O-methyl-beta-glucoside, cellobiose, gentiobiose, aesculin and salicin) and six polysaccharides (glycogen, hyaluronate, inulin, maltodextrin, pectin, and pullulan) (Table 4).

**Table 4:** Carbon sources utilised by *S. pneumoniae* DP1004.

		Phenotype microarray^a^	Fermentation^b^	Growth^c^	Doubling time (min)	μ^d^
C3 compounds	glycerol^e^	99	+	+/−		
C6 compounds	ascorbate	nd	+	+	640	0.06
	fructose	177	+++	+++	46	0.90
	galactose	153	+++	+++	61	0.67
	glucosamine		+++	+++	46	0.89
	glucose	158	+++	+++	32	1.28
	mannose	157	+++	+++	44	0.95
	GlcNAc	169	+++	+++	42	0.97
	ManNAc^e^	140	++	+	241	0.17
	NeuNAc^e^	93	++	++	121	0.32
alpha-galactosides	melibiose	152	+	+	169	0.24
	raffinose	165	++	+++	43	0.95
	stachyose	166	+++	+++	45	0.93
beta-galactosides	lactose	173	+++	+++	42	0.98
	lactulose	138	nd	+	282	0.14
alpha-glucosides	maltose	164	+++	+++	42	0.99
	maltotriose	166	++	+++	46	0.89
	sucrose	170	+++	+++	39	1.07
	trehalose	171	+++	+++	45	0.92
beta-glucosides	aesculin	nd	++	+	116	0.35
	amygdalin	171	+++	+++	72	0.57
	arbutin	nd	+	−		
	methyl-beta-glucoside	146	+++	++	90	0.46
	cellobiose	117	+++	+++	76	0.54
	gentiobiose	149	+++	+++	62	0.67
	salicin	-	++	++	93	0.44
polysaccharides	glycogen	-	++	+++	62	0.66
	hyaluronate	nd	++	+	139	0.29
	inulin	-	+	−		
	maltodextrin	nd	++	+++	38	1.09
	pectin	-	++	nd		
	pullulan	nd	+++	nd		

(a) phenotype microarray results are expressed as average height of reaction after subtraction of the background represented by culture medium. nd = not done, − = negative (below cut off for positivity).

(b) fermentation results are expressed as arbitrary scores evaluating the colour change of the pH indicator in CAT medium with 1% sugar, except for NeuNAc.

(c) growth is reported as arbitrary units for maximal cell concentration.

(d) growth, doubling time and the constant μ refer to growth in CAT medium with 0.3% sugar.

(e) glycerol, ManNAc and NeuNAc values were obtained from strain G54.

Amongst the different assays used, the analysis of the growth parameters (i.e. maximum cell concentration and maximum growth rate (μ) proved to be the most reliable to evaluate differences in metabolic substrates and the effect of mutations on substrate utilization. Comparison of growth rates showed that for strain DP1004 glucose was the sugar supporting the fastest growth (generation time of 32 minutes). In contrast N-acetylmannosamine (ManNAc), ascorbic acid and the beta-galactoside lactulose showed the longest generation times (Table 4). Overall the results obtained using fermentation, growth and phenotype microarray were consistent with the exception of polysaccharides, glucosamine (GlcN) and pentose utilisation, which could not be detected by phenotype microarray (Table 4). When using the commercial API 50 CH carbohydrate tests and the rapid ID 32 STREP identification system (both from bioMerieux), the results were in perfect accordance with our in house assays (data not shown).

The principal glucose transporter in *S. pneumoniae* is the mannose type PTS SP0282-3-4. Upon all mutants generated in this work, the deletion of this PTS was the only one to affect the utilization of glucose. Data from *S. salivarius* and *S. mutans* are in agreement with this mannose type PTS being the main glucose transporter the PTS responsible for carbon catabolite repression [94], [95]. In addition mutation of PTS SP0282-3-4 affected growth on mannose, galactose, GlcNAc, and GlcN when used as sole carbon source (Figure 2). The SP0282-3-4 mutant strain showed no differences in the final OD when grown on glucose, but an increased doubling time could be detected (47 min vs 32 min, p<0.001) (Figure 2D). The absence of a clear phenotype related to glucose utilization was thus probably to ascribe, as in most organisms, to a series of efficient glucose uptake systems. The deletion of the SP0282-3-4 PTS resulted in a total loss of growth in presence of GlcNAc (Figure 2A), indicating that this is probably the only pneumococcal transporter for this sugar, inasmuch pneumococci also lack the *nagE* PTS [96]. The SP0282-3-4 transporter was also found to be the system responsible for the uptake of the amino sugar GlcN. GlcN appeared to be toxic at high concentration such as 10 g/l, where wild type *S. pneumoniae* reached OD = 0.12, while at concentration of 3 g/l it grew until OD = 0.3 (Figure 2E). The mutant strain for this transporter grew to OD = 0.24 at GlcN of 10 g/l, while at lower, non-toxic, concentrations of 3 g/l and 1 g/l it exhibited a generation time increased by 35 min (81 min vs 46 min, p<0.05) and by 80 min (131 min vs 41 min, p<0.001) respectively. Growth was not totally abolished since another PTS system (SP1684) responsible for GlcN uptake could be identified. Deletion mutants for SP1684 showed a prolonged generation time on GlcN (56 min vs 46 min, p<0.05) (Figure 3E). The lack of the EIIA subunit in SP1684, led us to investigate double mutants of SP1684 with the other glucose type PTS systems, but no effects in the presence of the sole GlcN could be observed(data not shown), probably due to interactions of different EIIA subunits with this PTS system.

Galactose uptake could be traced to at least three transporters, the mannose type PTS SP0282-3-4 (see above), the SP0645-6-7 galactitol type PTS [56], the SP0090-1-2 CUT1 and probably the SP0061-2-3-4 mannose type PTS. The clearest phenotypes could be detected in SP0282-3-4 and SP0645-6-7 PTS deficient strains which showed a decreased final cell density and an increased doubling time in galactose, respectively increased by 26 minutes (87 min vs 61 min, p<0.005) in 0.3% sugar and 31 minutes (93 min vs 62 min, p<0.01) on 0.1% of galactose. The SP0645-6-7 mutant showed only a slight increase of doubling time when grown at 0.3% of galactose (Figure 2C). In addition to galactose, also lactose and galactitol had been reported to induce SP0645-6-7 [56] , albeit no growth on galactitol and no growth defect of the mutant in lactose could be observed by us (data not shown). A weak galactose phenotype was found for the SP0090-1-2 CUT1 ABC transporter mutant which showed reduction in sugar utilization in the phenotype microarray where a 15% reduction in redox potential generation was measured. Galactose, when imported by ABC transporters or permeases, is phosphorylated intracellularly at the C1 position by a specific kinase (GalK) and metabolised not by the tagatose-6-phosphate pathway, but by the Leloir pathway [97] . The necessary genes are not in a conserved operon structure in the diverse lactic acid bacteria [98]. In pneumococci the genes for the Leloir pathway are located in three distinct operons which are not linked to any ABC transporter. Phenotypic analysis of the mutant strain for SP0061-2-3-4 mannose type PTS by phenotype microarray, fermentation and growth assays did not show any difference to the wild type strain (data not shown). But, since the beta-galactosidase was characterized as a surface enzyme responsible for the cleavage of Galβ1-3GlcNAc [99], [100], we assume that SP0061-2-3-4 may be a further galactose uptake system.

The mannose type PTS SP0282-3-4, the lactose type PTS SP0476-8 and the SP0090-1-2 CUT1 could be related to mannose uptake. SP0282-3-4 had a very clear phenotype with a decreased final cell density and an increased doubling time (89 min vs 44 min, p<0.001) (Figure 2B), while SP0090-1-2, similarly to its phenotype in galactose, showed in the phenotype microarray a 25% reduction in the metabolic activity. While the single mutant for SP0476-8 showed no phenotype, the double mutant with SP0282-3-4 (Figure 2B) showed only residual growth on mannose, probably due to the activity of the SP0090-1-2 ABC transporter.

In addition to N-acetylglucosamine and glucosamine, pneumococci fermented also the amino-sugars N-acetylmannosamine (ManNAc) and N-acetylneuraminic acid (NeuNAc; sialic acid). Uptake of NeuNAc has been mapped recently to the SP1688-9-90 ABC transporter [28]. Our data here describe a more complex outline for amino-sugar uptake, which includes at least four transporters and both NeuNAc and its catabolic product ManNAc, generated in a single step by the NeuNAc lysase. Since D39 did not ferment sialic acid due to a frame shift in its lyase, mutants for phenotypic analysis were also constructed in strain G54. In both genetic backgrounds, growth on MaNAc was delayed by ten to twelve hours in the mutant deleted for the whole *nanAB* regulon (Figure 3F), which includes three transporters (Figure 1) [72]. The lag phase is probably due to absence of the SP0090 ABC transporter, whose mutant showed 25% reduction in ManNAc metabolism in phenotype microarray, and the two allelic alternative transporters in the NanC operon (see end of paragraph). Mutants for the single ABC transporters of the *nanAB* regulon always started to grow with a lag period when compared to the wild type strain (Figure 3F), however the ABC SP1681-2-3 mutant grew with a generation time comparable to G54, while the ABC SP1688-9-90 mutant was affected more severely (367 min vs 241 min, p<0.001) indicating that SP1688-9-90 is the more efficient ManNAc transporter. G54-derived mutants deleted for the whole *nanAB* regulon showed a complete absence of growth on NeuNAc (Figure 3G). This was remarkable since also mutation of the *nanC* operon containing the SP1328 sodium-solute symporter also affected NeuNAc metabolism (phenotype microarray 40% reduction). To explain the lack of growth of the *nanAB* mutant in sialic acid we hypothesize that some of the enzymes present in *nanAB* are essential for sialic acid utilization. In both the SP1688-9-90 and SP1681-2-3 ABC mutant growth of strains was affected with an increase in the generation time by 30% and 50% respectively (158 min and 179 min vs 121 min, p<0.05 and p<0.01) (Figure 3G), which is in accordance with the identification of SP1681-2-3 as the main sialic acid transporter [28]. When grown on ManNAc, pneumococci showed a longer generation time compared to growth on most other carbohydrates, including NeuNAc. These data indicate that ManNAc is probably transported by these two systems at lower efficiency than NeuNAc. The reduction of ManNAc and NeuNAc metabolism in mutants of the *nanC* operon, containing the SP1328 sodium-solute symporter was 20% and 50% respectively when assayed by phenotype microarray. Also deletion of the corresponding operon in DP1004, carrying the spr1191-2-3-4 ABC transporter, lead to a 20% reduction of ManNAc metabolism in phenotype microarray. A 25% reduction of ManNAc metabolism was in addition observed in the mutant SP0090-1-2. The fact that reduced growth and/or metabolism of NeuNAc and ManNAc was observed in mutants for so many transporters indicates a complex metabolic and genetic regulation [9].

Even though five PTS transporters belong to the lactose type family, only the SP1185 PTS mutant has shown a phenotype related to lactose uptake. Knock out mutant for the genes encoding this transporter had a 20 minutes increase in the doubling time on this substrate (63 min vs 42 min; p<0.01) (Figure 3A) and showed a complete loss of growth in the presence of lactulose (Figure 2R). In *S. mutans* the orthologous PTS (smu.1492) has been shown to be induced by lactose and galactose [69].

**Figure 4:**
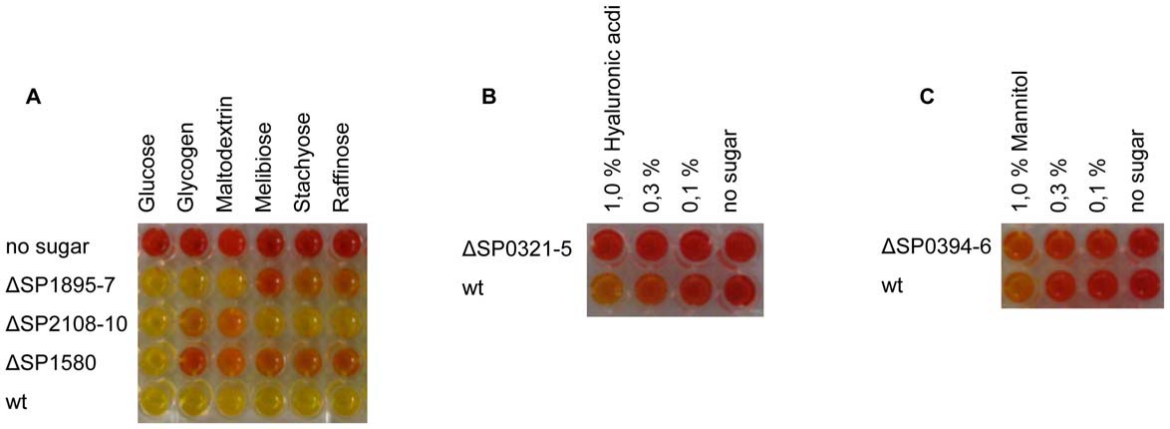
Fermentation assays. Acid generation from sugar substrates is indicated by colour change (yellow) of the pH indicator phenol red after incubation for 24 hours. (A) Negative control, ΔSP1895-6-7, ΔSP2108-9-10 , ΔSP1580 and wild type in presence of 0.3% of glucose, glycogen, maltodextrin, melibiose, stachyose and raffinose; (B) wild type and ΔSP0321-3-4-5 in serial dilutions of hyaluronic acid; (C) wild type and ΔSP0394-6 in serial dilutions of mannitol.

In standard conditions pneumococci are unable to use mannitol as a carbon source [101]. This is also reflected by all commercial identification systems, which list pneumococci as mannitol negative. A weak mannitol-dependent acidification of the medium could be evidenced when incubating pneumococci at double density with respect to standard fermentation assays. Deletion of the SP0394-6 galactitol type PTS without affecting *mtlD* reduced acidification of the medium after 24 hours from pH 5.7 by the *wt* to pH 6.2 by the mutant (fresh medium at pH 6.8) (Figure 4C). This suggests that SP0394-6 is the mannitol PTS of pneumococci and that another transporter appears to be able to import this polyol. Interestingly, strains lacking the entire operon, and thus also the mannitol-1-phosphate 5-dehydrogenase MtlD, were completely negative for mannitol fermentation.

Deletion of the SP0877 fructose type PTS resulted in complete loss of fructose fermentation without any detectable effect on other sugars (Figure 2Q). In contrast to *L. lactis*, the mannose PTS appears to have no specificity for fructose, thus leaving the fructose PTS the only transporter for this ketose [102].

The metabolic assays conducted on the SP0321-3-4-5 mannose type PTS mutant showed reduced fermentation (Figure 4B) and a total lack of growth in hyaluronic acid (Figure 2K). Since *S. pneumoniae* is unable to utilize glucuronic acid (GlcU) (data not shown) and uptake of GlcNAc is performed exclusively by the PTS SP0282-3-4 (Figure 2A) we speculate that the hyaluronate disaccharides are the substrate for this transporter due to the presence in this locus of an extracellular hyaluronidase and intracellular SP0322 hydrolase for which the substrates had been defined [49]. No further assays have been performed on other glycosaminoglycans. Annotations both in *S. pneumoniae* and *S. pyogenes* describe this PTS as an N-acetyl-galactosamine transporter, but in all conditions assayed in this study pneumococci were unable to use GalNAc as a carbon source.

**Figure 5:**
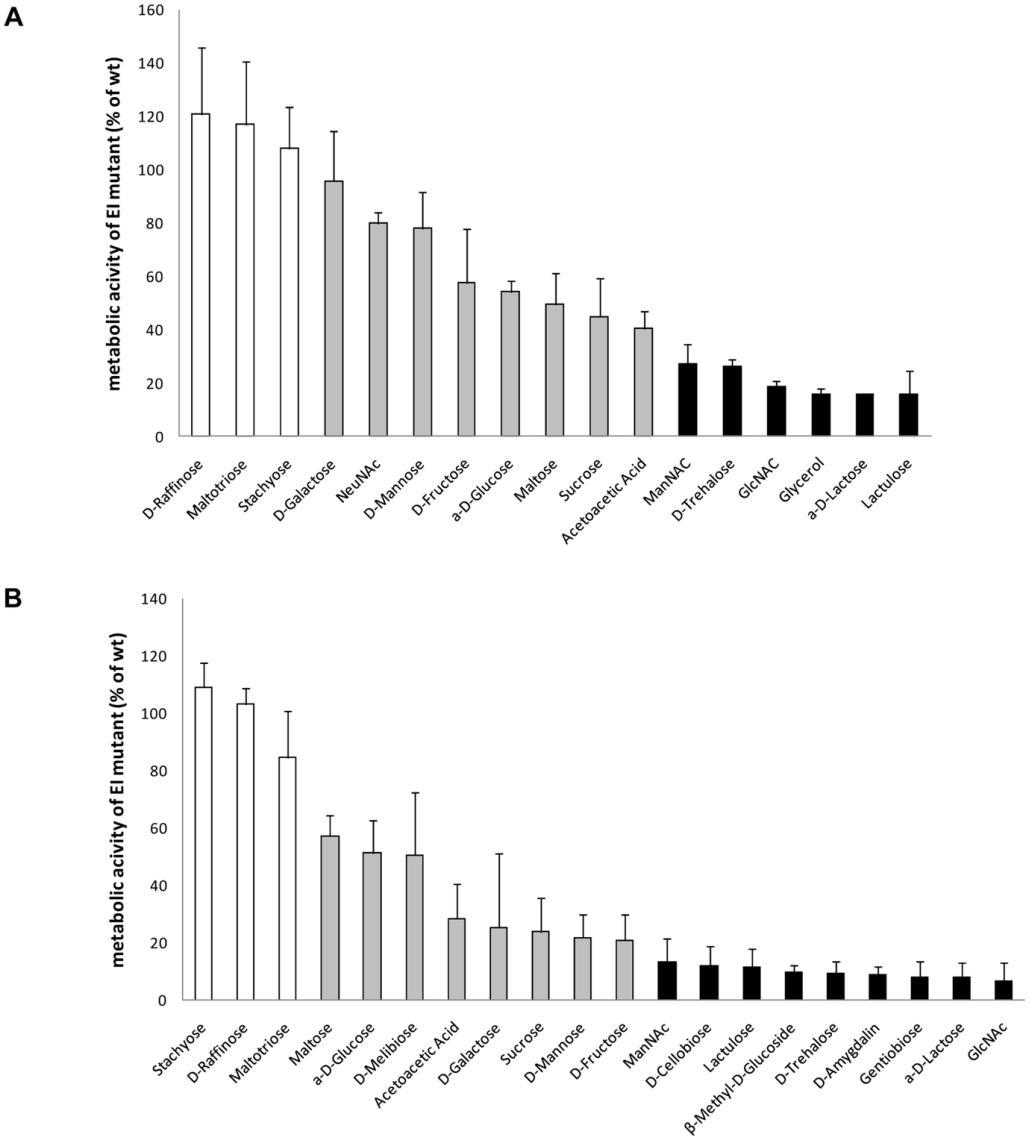
Carbon source utilisation profiles of EI mutants. The relative metabolic activity of EI mutants of G54 (panel A) and DP1004 (panel B) was determined by phenotype microarray. Relative metabolic activity is expressed as percent of the mean metabolic activity of the mutants with respect to the wild type using triplicate experiments (Biolog Omnilog-PM software parameter average height). Shading refers to carbon sources whose uptake is not influenced (white bars), influenced in part (grey bars) or abolished (black bars) by mutation of *ptsI*.

Contribution to maltose and malto-oligosaccahrides uptake depended on the length of the polymers, with the SP0758 PTS being responsible for the transport of short sugars and the SP2108-9-10 ABC transporter for longer oligosaccharides. Deletion of the *malXCD* SP2108-9-10 CUT1 ABC transporter resulted in reduced final cell density and increased doubling time (131 min vs 62 min, p<0.001; 111 min vs 38 min, p<0.005) in the presence of the polysaccharides glycogen and maltodextrin (Figure 2O, 2P). Deletion of the *msmK* gene SP1580 showed the same phenotype as the SP2108-9-10 mutant (Figure 2O, 2P). A double mutant for *msmK* and the SP0758 glucose type PTS abolished completely growth on these polysaccharides (Figures 2O, 2P). The single SP0758 PTS mutant strain had no phenotype on this two branched polysaccharides (Figure 2O, 2P), indicating that its malto-oligosaccharide transport is less efficient. The use of trisaccharide maltotriose showed no reduced uptake in any of the single PTS and ABC mutants while in the double SP0758 - SP1580 mutant, growth was totally abolished (Figure 2N). The knock out mutant of SP0758 PTS resulted in reduced growth on maltose (50% reduced final cell density, Figure 2M), indicating that SP0758 is not the only uptake system for maltose. The fact that in the EI mutant maltose metabolism was only reduced (Figure 5A, 5B), indicated that part of the maltose transport should be due to a non-PTS transporter. The double mutant strain for this PTS transporter and *msmK* (SP1580) showed a growth behaviour on this disaccharide comparable to that of the single PTS mutant. This last fact suggests that in addition to the SP0758 PTS at least one other pneumococcal transporter is involved in maltose import.

The two sucrose transporters were characterized by Iyer and Camilli [27] who kindly provided us their mutant strains. Our phenotypic assays confirmed the previous results and showed that deletion of the SP1722 glucose type PTS resulted in growth in sucrose with an increased doubling time (121 min vs 39 min, p<0.001), while the mutant strain for the SP1796-7-8 CUT1 ABC transporter alone had no effect in our genetic background (Figure 3B). The double mutant for both SP1722 and SP1796-7-8 lost completely the capacity to grow on sucrose (Figure 3B) confirming published data [27].

The operon of the SP0305-8-10 lactose type PTS had been shown to be responsible for the metabolism of beta-glucosides [103]. As discussed above all pneumococcal strains that fermented cellobiose and gentiobiose carry this locus, while all strains lacking this locus do not ferment cellobiose and gentiobiose. This is confirmed by fermentation and growth assays carried out with the R6 spr0282 EIIC mutant (provided by Regine Hakenbeck), which lost the capacity to metabolise these two sugars. Testing additional beta-glucosides confirmed uptake of arbutin (tested only in fermentation assay, Table 3), amygdalin and aesculin by this operon. Growth on all these substrates was almost totally abolished in the mutant of this transporter (Figure 2F, 2G, 2H), with the exception of aesculin where cell density reached half of the maximal OD (Figure 2I). Two other transporters were identified for beta-glucosides, the SP0577 glucose type PTS, whose deletion led to a doubled generation time (195 minutes) and a prolonged lag period (Figure 2J) on 1-O-methyl-beta-glucoside and the SP2022-3-4 lactose type PTS, whose deletion caused a 20% increase of the generation time (87 min vs 72 min) and a reduced cell density (Figure 3H) on amygdalin. Furthermore, growth of the SP2022-3-4 knock out mutant on cellobiose extended the lag period by 2 h. The finding that the SP0305-10 PTS mutants failed to metabolise cellobiose and amygdalin appears to be in contrast with these findings, indicating the need for further work to elucidate interconnections between the three systems. Lack of fermentation of the beta-glucoside salicin, evidenced in some pneumococcal strains (Table 3), could not be shown for any of the three beta-glucoside transporter mutants.

In the fermentation assay, acidification of the medium were analysed for ascorbate concentrations only up to 0.5% w/v. The mutant strain for the SP2036-7-8 asc-type PTS system showed a limited reduction in growth on ascorbate (Figure 3E). The fact that growth was not totally abolished indicates that another uptake system is capable to transporting this compound, possibly the other asc-type PTS system present in pneumococcus SP2129-30. Fermentation assays in the presence of ribose, xylose, arabinose and xylulose, have been carried out for up to 5 consecutive days, since pentose utilization is described as very slow [10], but only unspecific medium acidification could be detected even in control samples. Since the transketolase present in the SP2129-30 operon catalyzes the conversion of xylulose-5P in fructose-6P, we propose this PTS to transport ketopentoses, such as xylulose or ribulose. Another predicted transporter for pentoses is the SP1617-8-9 fructose type PTS. No mutant for this PTS was generated so far, since the D39 derivative used for mutagenesis did not contain the operon. A mutant strain for the genes for this PTS was constructed in TIGR4, but no phenotype could be detected for pentoses, since the wt did not grow on ribulose [104]. In *S. mutants* the SP0845-6-7-8 CUT2 ABC transporter orthologue is reported to transport ribonucleosides [79], but our pneumococci failed to utilise this carbon source. Also the assay for sensitivity to 5-fluorouridine, performed as described for *S. mutans* [79], failed in pneumococci, since our strains were not sensitive to this toxin (data not shown).

Pneumococci have two glycerol facilitators, *sensu strictu* glycerol facilitator SP2184 and a second putative glycerol facilitator SP1491. In R6, but not in its encapsulated progenitor D39, *glpD* is frame shifted. The absence of a functional *glpD* would be expected to block the utilization of glycerol, via glycerol 3-phosphate and glycerone phosphate. In the biolog, the low metabolic activity detected for glycerol in strains TIGR4 and G54 was completely absent in the D39 derivative DP1004, which shares R36A as a progenitor with R6 [105]. The same observation was made for growth in liquid medium where DP1004 was not able to grow in presence of the glycerol compared to G54 and TIGR4 (Figure 3K). The *ptsI* mutant did not metabolize glycerol in phenotype microarray assays, although glycerol is not transported by the PTS (Figure 5). This is explained by the fact that glycerol kinase of *Firmicutes* is phosphorylated by phosphoenolpyruvate, EI and HPr and that this modification is necessary for the activation of the enzyme and hence metabolic use of glycerol [106]. SP1491 is, in addition to SP2184, the second pneumococcal MIP family transporter (TC1.A.8) annotated as a putative glycerol facilitator. Many species carry multiple MIP family transporters, and often more than one is annotated as glycerol facilitator. In *L. monocytogenes* deletion of either one of the two glycerol facilitators alone had no effect on glycerol metabolism [107]. No mutant for this transporter was constructed in this work and no phenotype associated. Standard disk diffusion assay for antimicrobial susceptibility testing using phosphomycin yielded the same inhibition zones of 22 mm for D39, R6 and DP1004. Such unaltered susceptibility to phosphomycin, a commonly used antibiotic which enters the cell via GlpT [92], indicates that the operon in R6 is still expressed and the transporter is still functional despite the *glpD* frame-shift.

Deletion of SP1884 glucose type PTS resulted in impaired growth in trehalose (Figure 3E). The mutant strain for the SPH1925-6-7 and SPH1929 lactose type PTS in G54, due to absence of this PTS in DP1004, showed no phenotype when assayed in phenotype microarray. Considering the annotation of the surrounding genes, the operon appears to be involved in the uptake of some sulphated carbohydrates and concomitant export of some (toxic) metabolic derivatives thereof [108]. The SP2161-2-3-4 Mannose-Fructose-Sorbose type PTS and the SPG2105 CUT-1 ABC transporter present in some strains in the same location are part of an operon containing enzymes for fucose utilization, but since pneumococci do not grow on fucose, the transport of fucose and fucose di and oligosaccharides could not be confirmed in our assays [104]. Alpha-galactosides are transported by the SP1895-6-7 CUT1 ABC transporter in accordance with the report showing induction of its promoter by raffinose [25]. In our assays the mutant strains lacking either SP1895-6-7 or the *msmK* gene, became unable to grow in the presence of raffinose, stachyose and melibiose (Figure 3H, 3I, 3J).

### Central transporter regulation

#### SP1176 Enzyme I

We evaluated the global impact of PTS transporters on sugar uptake by analyzing pneumococci mutated in EI (enzyme I, *ptsI*, SP1176). Data were collected by phenotype microarray of EI mutants in both D39 and G54 backgrounds (Figure 5A, 5B). As shown in Figure 5A and 5B, uptake of alpha-galactosides and maltotriose does not decrease in the *ptsI* mutants of both strains indicating that the contribution of PTS systems is either absent or negligible. A group of compounds showed a partially reduced metabolism, and thus most probably these sugars are transported in pneumococci by both a PTS and a non-PTS transporter. These sugars include maltose, glucose, galactose, sucrose, mannose and fructose. Amongst the sugars whose metabolism was completely blocked in the *ptsI* mutant, there were the beta-glucosides, trehalose, GlcNAc, lactose and lactulose, glycerol only in G54 and surprisingly ManNAc.

#### SP1580 CUT1 ATP-binding cassette domain protein

MsmK (SP1580) encodes the sole ATP-binding cassette domain protein of the CUT1 family [22]. A mutant of SP1580 was assayed in fermentation and growth assays and in phenotype microarray. In all assays the phenotype of the SP1580 mutant overlapped the combined phenotypes of the CUT1 transporters SP1895 for alpha-galactosides and SP2108 for polysaccharides (Figure 2O, 2P, 3H, 3I, 3J, 4A), while none of the phenotypes associated with the mutants for the other CUT1 transporters SP0090-1-2 (galactose, mannose and ManNAc), SP1681-2-3 and SP1688-9-90 (NeuNAc and ManNAc) and the SP1796-7-8 (sucrose), could be detected in SP1580. These data are in part in accordance with work in *S. mutans*, where the MsmK and MalK ATPase subunit proteins are able to interact either with their own or with an alternative transport complex, but in discordance with its involvement in sialic acid transport [23], [109].

**Figure 6:**
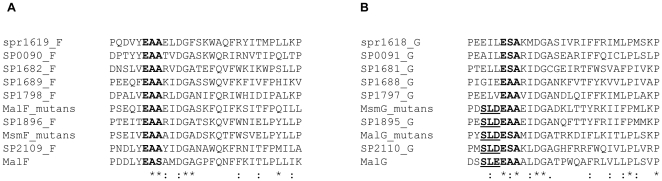
Alignment of the EAA loop region of the CUT1 ABC transporter permeases. The two permeases forming the heterodimer of CUT1 uptake systems are aligned to the reference MalF and MalG proteins of *E. coli* respectively in panel A and panel B. The EAA loop regions represent the domains of interaction between the permeases with the ATP cassette binding proteins [136]. Sequences aligned correspond to amino acid 396 to 424 of MalF and 185 to 213 of MalG of *E. coli*. Sequences aligned are from all six pneumococcal CUT1 permeases of TIGR4, the spr1618-9 allele of R6 for SP1797-8, the SPG2105-6 G54 allele for the PTS SP2161-4 and the two CUT1 permeases MalFG and MsmFG of *S. mutans* UA159. The EAA triplet is in bold and the SLD consensus is bold underlined.

In order to search for a molecular explanation for the selective interaction of SP1580 with the permeases SP1895-6 and SP2109-10, we tried to identify specific conserved sequence motifs. Interaction between the subunits of the transporters have been mapped to the so called EAA domain of about 20 amino acids located in the second to last trans-membrane domain of the permeases and to the Q-loop of the ATPases [110], [111]. Alignment of the MalG-like permeases revealed a conserved sequence motif of three amino acids within the EAA domain. The amino acid triplet S L D precedes the conserved E A A triplet and is present, not only in the two pneumococcal proteins SP1895 and SP2110 and in the *S. mutans* MalG and MsmG, but shows very high homology to the *E. coli* MalG (Figure 6B). No such consensus could be found in the MalF-type permeases (Figure 6A). To explain the functionality of the remaining ABC transporters we analysed more than 70 pneumococcal proteins with an ABC-cassette domain typical of the ABC transporter ATPases. Alignment of the ATPases, or selectively of the Q-loop of the ATPases, indicated that three proteins cluster with SP1580 (i.e SP0242, SP1389, and SP1825). Still, the *E. coli* MalK was more similar to SP1580 than any of the other ATPases, indicating that their interaction with CUT1 proteins is improbable. The identity of the ATP-binding cassette protein driving import by SP0090-1-2, SP1681-2-3, SP1688-9-90 and SP1796-7-8, is thus in our view, not solved [23].

## Discussion

### Genomic analysis of transporters in *S. pneumonia*

In the present work we performed a genome wide analysis of all sugar transporters present in *S. pneumoniae*. These fall into four classes of established transporter types, including channel proteins (TC 1.A.), secondary active transporters (TC 2.A.), primary active transporters (TC 3.A.), and group translocators belonging to the sugar-transporting phosphotransferase system (PTS) (TC 4.A.). Out of a total of thirty sugar transporters identified in twenty-six sequenced pneumococcal genomes, the PTS transporters are between fifteen and twenty per genome, with a total of twenty-one PTS systems identified (Figure S1), seven to eight primary active transporters of the ABC transporter super family and only one member for the channel protein family and the secondary active transporters. This general genomic pattern of transport proteins in the pneumococcus is in accordance with other bacteria lacking the tricarboxylic acid cycle, and generating energy by substrate level phosphorylation. These bacteria, that utilize ATP as the sole energy source, have mainly ATP driven primary pumps and group translocators and lack channels and electrochemical driven transport [37]. Still, the number of PTS systems and carbohydrate ABC transporters is exceptionally high with respect to the genome size of pneumococci. Such abundance of sugar transport systems places the streptococci, together with clostridia and enterococci, amongst the species with the highest concentration of carbohydrate uptake systems [12], [37].

### Comparative genomics and phenotypes

Comparative genome analysis had already shown that a significant part of genomic variation in pneumococci is due to carbohydrate uptake systems and metabolically related operons [29]–[31]. The high number of carbohydrate transporters previously described outnumbers the substrates described so far for *S. pneumoniae* [112]. In order to better characterize the origin of genomic variation and to fill a gap in the insufficient annotation of transporters, we assessed the metabolic activity by determining the overall carbohydrate metabolic profile of pneumococci, using fermentation assays and metabolic phenotype microarray. The data obtained reveal that *S. pneumoniae* is able to utilize at least thirty-two substrates (Table 4) as carbon sources, including sugars like mannose, galactose, GlcNAc and NeuNAc, mainly found in mucin O-glycans and human cell surface N-glycans, and hyaluronic acid, found also on the apical surface of epithelial cells of the airways [113], but also many food-borne monosaccharides, disaccharides (alpha- and beta-glucosides and alpha- and beta-galactosides) and polysaccharides. This is a hallmark of bacteria adapted to ecological niches providing a wide variety of carbon sources, such as the oral cavity and the nasopharyngeal mucosa which contain food- and host-derived sugars. The absence of some types of sugars from human tissues or alimentary sources should not rule out their metabolic use by pneumococci. In fact, the human microbiome is large and for example enzymes like the trehalose synthase is readily detected when searching in human oral metagenomic datasets (http://www.homd.org/).

Very challenging was the identification of some sugars, which are weakly metabolised by *S. pneumoniae*, such as glycerol (Figure 3K) and mannitol (which required a double density inoculum in the fermentation assay). Slow acid production from glycerol, xylose, arabinose and erythritol is also reported for pneumococci in Bergey's Manual [10], but metabolic activity could not be detected for the latter. We have no justification for such a weak metabolism and long generation time. It must be noted that all assays used in this work are metabolic assays and do not detect directly carbohydrate uptake. Our assays thus miss any carbohydrate uptake that would not feed into carbon dependent energy metabolism. An example for this is fucose, which cannot be metabolized, since genes required for fucose/lactaldehyde metabolism are missing (www.genome.jp/kegg/). However, a complex fucose operon is present in two allelic forms, and the substrate binding of its ABC transporter had been defined as being specific for the terminal residues of blood group antigens [78]. A non-metabolic use of fucose has been described for *Bacteroides*, where this sugar acts as a regulator of other operons and is utilised for synthesis of oligosaccharides [114].

### Substrate identification of transporters

In order to correlate the genome variability of sugar transporters with carbohydrate consumption we analyzed 26 sequenced pneumococcal strains for fermentation of 20 different carbon sources. Despite the high variability of transporters and the large number of strains we were able to correlate a phenotype only for two transporters. With the hindsight of data accumulated during this work, the frequent substrate redundancy amongst some transporters may explain the low success rate of this approach. Transporters possibly identified by using exclusively a quantitative assay, such as the fermentation, may include the main beta-glucoside operon SP0305-8-10 [103], the trehalose PTS SP1884, identified by us, the ABC transporter SP1895-6-7 for alpha-galactosides and the PTS SP0877 for fructose. While the first two transporters were readily identified, the other two were not, since conserved in all strains (Table 2).

Mutant construction in all carbohydrate uptake systems permitted us to identify the substrate specificity for most of the 30 transporters. For those sugars which were inefficiently metabolized (i.e. glycerol, arbutin, mannitol) fermentation proved to be the only suitable assay, while for those substrates more efficiently metabolized, analysis of growth kinetics was the most sensitive method. In some cases, construction of double mutants was required, due to uptake of the same substrate by different transporters, which could often be compensated in single mutants. An example is the SP0476-8 PTS system, which remained uncharacterized until a double mutant with the broad-substrate PTS SP0282-3-4 was generated (Figure 2B). In this case growth on mannose, affected in the SP0282-3-4 single mutant, but not in SP0476-8, was almost totally abolished in the double mutant. A similar situation was described for the two sucrose uptake systems already described by Iyer and Camilli, where the lack of SP1796-7-8 ABC transporter became only perceivable in the double mutant with the PTS1722 [27] (Figure 3B).

For some transporters without a phenotype, a possible substrate candidate could be identified only through comparison with orthologues in other organisms. The SP0061-2-3-4 PTS system could be assigned to galactose uptake since a surface Galβ1-3GlcNAc beta-galactosidase (BgaC) [99], [100] and other enzymes for galactose utilization are present in the operon [115]. No phenotype was observed in our mutants for this transporter probably due to the presence of at least three other galactose transporters. The SP0845-6-7-8 CUT2 ABC transporter has over 70% identity to the orthologue operon in *S. mutans*, which was found to transport ribonucleosides [79]. The inability of pneumococci to ferment these compounds and the absence of a phenotype in the indirect toxicity assay with 5-fluorouracil did not allow experimental validation of our mutant. SP1617-8-9 and SP2129-30 PTS systems are predicted as pentose and ketopentose transporter, but also in this case the absence of fermentation of five-carbon sugars did not permit experimental validation. Similarly, *S. pneumoniae* is not able to use fucose as a carbon source, and none of our assays could be used for the analysis of the two alternative alleles of transporters SP2161-2-3-4 PTS and SPG2105-6-7 CUT-1 ABC of the fucose operon [77], [78]. Uptake of radiolabelled substrates may allow experimental demonstration of some substrate specificities. Only for two PTS systems we could not identify any substrate, one being the SP0248-9-50 PTS and the other SPH1925-6-7 PTS. The first, conserved in all the sequenced pneumococcal strains, lies in an operon coding also for a pyruvate formate lyase, suggesting a role in the mixed-acid fermentation, but, even though the orthologue of this transporter has been found in many other bacterial species within identical operon structure, none of them have been characterized so far. SPH1925-6-7 is a lactose type PTS found in half of our sequenced strains and encoding genes for two separate EIIC genes on the same indel. This PTS, whose orthologue is present in only a few other species, is part of a metabolic operon which seems to be involved in uptake of sulphated sugars, such as those present in human mucin [116], with subsequent removal and export of the sulphite by a cotranscribed efflux system of the TauE family [108].

In some cases minor differences with previously published work were noticed. Mainly this was not due to the type of substrate assigned, but related to the extent of phenotype detected in certain mutants, i.e. a SP1688-9-90 mutant, which grew well on sialic acid [28], a SP1580 mutant which did not grow in sialic acid [23] and a SP1796-7-8 mutant which exhibited reduced growth in sucrose [27]. In all three cases we obtained in our assays a different result, which in the case of SP1580, led us to a different conclusion. This underlines the fact that growth parameters are complex markers, which are heavily influenced by other factors including genotype of strain, composition of the media, inoculum size and composition, substrate induction, polar effects of mutants, loss of potential regulatory effects mediated by the transporters themselves or by the regulators of the mutated loci. These facts are difficult to control and should remind us that, especially in the presence of multiple uptake systems with redundant specificities, some conclusions of this work are amendable to be revised by further work.

### Generation time

Other than being a powerful tool for identification of transport specificity, the determination of generation time gave us insight into pneumococcal growth on substrates different from glucose. This is an important issue since in vitro growth assays are usually performed in the presence of glucose, that is scarce in the mucosa (Philips *et al.*, 2003). It is striking to note that generation times span from 31 minutes, for the most efficient sugar glucose, which is abundant only in blood, to the longer generation times of sugars available in mucus or tissue as GlcNAc (42 min), mannose (44 min), galactose (61 min), sialic acid (128 min) or hyaluronic acid (139 min). Understanding pneumococcal growth on different natural substrates may help finding a correlation between carbon source availability and pathogenesis. It is tempting to suggest that our data indicate that pneumococci may in fact replicate very rapidly in the blood, while being somewhat hampered on mucosal surfaces, not due to some unknown mechanism, but due to intrinsic regulation of their carbohydrate metabolism. In the human host, which is the sole known natural environment of the pneumococcus, most carbon sources are available as subunits of complex oligosaccharides. This is evident by the presence of extracellular glycosyl hydrolases in many of the operons analyzed, which, even though cleaving different glycan structures, often produce the same monosaccharides to be transported. It should be noted that the substrates of the transporters may often only represent the subunits of complex oligosaccharides which are metabolised by the entire operons (i.e. sulfated glucuronic acid disaccharides, complex alpha-galactosides, different sialic acid oligosaccharides) [49], [51], [100], [117].

### Central control of PTS and ABC sugar transport

A remarkable feature of the large series of pneumococcal carbohydrate operons is that, not only the PTS transporters, but also the six CUT1 ABC transporters appear to be energised by a single protein. Such post-transcriptional central control adds to the complexity given by the many sugar binding and PRD containing transcriptional regulators and the regulation by CcpA on the *cre* sites [25], [56], [118], [119]. In this work we have reported the effect of deletion of both EI (Figure 5A, 5B) and MsmK (Figure 4A) in pneumococci. The presence in pneumococci of a single ATP binding cassette protein for a series of CUT1 permeases is similar to both *S. pyogenes* and *S. gordonii*, where genome data also indicate the presence of a single CUT1 ATP binding cassette protein together with permease/substrate-binding systems. The phenotypes observed in the pneumococcal *msmK* knock out mutant were consistent in all assays, but correlated only with phenotypes associated to alpha-galactoside (SP1895-6-7) and malto-oligosaccharide transport (SP2108-9-10) [25], [84]. This suggests that one or multiple ATP-binding-cassette proteins of the pneumococcal genome can provide energy for uptake of ManNAc, NeuNAc and sucrose by their respective permeases. This central role in metabolism of MsmK is also confirmed by the fact that this gene is detected in many genome wide assays for virulence determinants [120], [121]. Since the ATP binding proteins, MalK and MsmK of the orthologue transporters in *S. mutans*, were demonstrated being functionally interchangeable [109], we looked for a molecular signature of such an exclusive interaction. Alignment of the region of interaction of the permeases (EAA motif) to the ATP-binding proteins showed a possible three amino acid consensus preceding the EAA domain of the MalG-like permeases [110], [111]. The amino acid triplet SLD (Figure 6B), which was found in the SP1895-6-7 and SP2108-9-10, and in MsmG and MalG of *S. mutans*, also shows high similarity to the MalG protein of *E. coli*, but is not found in the other pneumococcal CUT1 permeases (Figure 6B). Out of all possible alignments, only the SLD consensus may provide a possible molecular signature for an exclusive interaction of the pneumococcal RafGF and MalCD permeases with the MsmK protein. We have no explanation why our results in G54 and DP1004 differ from a recent work, showing responsibility of MsmK in sialic acid uptake [23]. The most simple explanation could be differences due to the genotype of the strain used in that study [23].

To evaluate the global impact of all PTS systems on sugar consumption, we tried to disrupt the *ptsI* and *ptsH* genes, but only a *ptsI* mutant was generated, since *ptsH* seems to be an essential gene in pneumococcus [122], contrarily to *L. lactis* [123]. Since this is the only protein capable to initiate the PTS phosphorylation cascade from PEP, it is predicted to block sugar uptake by all the PTS systems, thus revealing the full set of this type of transporter. Mutation in D39 and G54 showed similar phenotypes in accordance with substrate specificity of the PTS transporters. Exceptions are partial reduction of fructose metabolism, even if only a PTS transporter was identified and reduction of NeuNAc and ManNAc transport even if these latter sugars are transported by ABC transporters and the sodium symporter. In the former case we hypothesize that, even if mutants of PTS0877 failed to grow in fructose, one ABC transporter could mediate fructose transport. In the case of the amino sugars it is more likely that the regulon, which has a *cre* site in front of each transcriptional unit, is regulated negatively by some P-Ser-HPr mediated mechanism of catabolite repression as described for *Lactobacillus casei* and *L. lactis* [124], [125]. It is of notice that, in contrast to MsmK, EI and HPr are generally not detected in screening for virulence determinants [120], [121], [126], since standard assays generally compare *in vivo* data to *in vitro* growth (on glucose) where EI/HPr play an important role, while MsmK does not.

In summary the present work represents the first genome wide analysis of carbohydrate systems in a bacterial strain carrying a large number of transporters. Data provide a global overview of all these systems, and identify substrates for almost all transporters and for over 80% of the substrates the appropriate transporter. In addition to the twenty-three experimentally confirmed transporter specificities, we could assign a function by genomic comparisons to further five transporters with the putative substrates being galactose, pentoses, ribonucleosides and sulphated glycans. Only for one PTS, possibly linked to mixed acid fermentation, we could not assign any substrate. This work is intended to guide re-annotation of genomic data and provides a detailed outline for functional analysis of carbohydrate metabolism in related Gram-positive species. In view of the important impact of many of the carbohydrate uptake systems and the related glycosyl-hydrolases in pneumococcal virulence, this detailed analysis provides the basis for the evaluation of the role of these systems in pneumococcal physiology during carriage and the regulation of progression to invasive disease.

## Materials and Methods

### Bacterial strains

Pneumococcal strains for comparative genomic and phenotypic analysis were the serotype 2 strain D39 [105] and its rough derivative DP1004 [127], the serotype 4 strain TIGR4 [12] and the serotype 19F strain G54 [14]. G54 is a serotype 19F strain resistant to erythromycin and tetracycline and was described as being highly competent for natural transformation [128]. The genome sequence of G54 has been completed by us by reassembling a previous unfinished genome [14]. The complete sequence has been deposited as NC_011072. All gene numberings refer to the genome sequence of strain TIGR4 (NC_003028). Pneumococcal strains with sequenced genomes and utilised recently for an extensive *in-silico* comparative genome analysis [32] were AP200 (kindly provided by Annalisa Pantosti, Istituto Superiore di Sanità Roma), 670-6B, CDC1087, MLV-016, SP195, CDC1873, CDC288-04, CDC3059-06 (kindly provided by Susan Hollingshead, Univ. Alabama), INV104B, INV200 (kindly provided by Tim Mitchell, Univ. Glasgow), SP11-BS70, SP14-BS69, SP18-BS74, SP19-BS75, SP23-BS72, SP3-BS71, SP6-BS73, SP9-BS68 (kindly provided by Fen Hu, Allegheny-Singer Research Institute) [129] JJA (kindly provided by Joice N. Reis, Oswaldo Cruz Foundation, Brazil), P1031 (kindly provided by Gerd Pluschke, Swiss Tropical and Public Health Institute, University of Basel, Switzerland), 70585 (kindly provided by Samir Saha, Dhaka Shishu Hospital, Bangladesh), SP195, 19A Hungary and 19F Taiwan (kindly provided by the Pneumococcal Molecular Epidemiology Network, PMEN) [32]. Strain identity was confirmed by sequencing a single MLST locus (*aroE*). Sequence data from these genome projects were retrieved from GenBank, from the Sanger web page and the Sybil multi genome comparison web tool.

### In silico analysis

Genomic comparisons and data analysis were performed using the following online tools, including the SYBIL multi-genome comparison tool [130], the BLAST at the NCBI and Sanger web sites (www.ncbi.nlm.nih.gov/BLAST/ and http://www.sanger.ac.uk/), and the Kyoto Encyclopedia of Genes and Genomes (www.genome.jp/kegg/) for metabolic pathway analysis. In house genome visualizations and alignments were performed using ATC and Artemis software from the Sanger Institute (www.sanger.ac.uk/resources/software/) [131] and BioEdit for BLAST analysis (http://www.mbio.ncsu.edu/BioEdit/).

### Mutant construction

Isogenic mutants were constructed by gene SOEing as already described [132], [133]. Primers for amplification were designed on TIGR4 sequences, and DNA constructs were transformed into the rough strain DP1004 a derivative of R36A, also parental of strain R6, which in turn derives from the serotype D39 [105]. Additional mutants for the *nanAB* locus (frame shift in the lyase gene in R6) and for the SPH1925 PTS (absent in R6) were transformed into a rough derivative of G54 [105]. The mutants for the sucrose uptake systems were kindly provided by Andrew Camilli (Tufts University) and those for the beta-glucoside EIIB by Regine Hakenbeck (Univ. Kaiserslautern). Primers for mutant construction that allow precise mapping of the deletions are in Table S1.

### Carbohydrate fermentation

To evaluate sugar uptake and fermentation the production of acid from sugar was determined. Bacteria grown on TSB blood-agar plates (Tryptic Soy Agar; Beckton Dickinson) were resuspended to an OD_590_ = 0.6 in CAT medium and diluted 1∶1 with CAT medium (Bacto Casitone 10 g/l, Bacto Tryptone 10 g/l, Yeast Extract 1 g/l, NaCl 5 g/l, K_2_HPO_4_ 0,5 M 30 ml/l) containing 0.6% sugar (0.3% sugar final concentration) and 200 U/µl of catalase. Aliquots were distributed into microtiter wells and incubated for 24 hours at 37°C. After incubation wells were coloured with phenol red (0.1 mg/ml) (P4633 Sigma-Aldrich). Fermentation was scored as positive if phenol red changed to orange or yellow, indicating a pH lower than 6.8. The API 50 CH and rapid ID 32 Strep identification systems were used according to the instructions provided by the supplier (BioMerieux, La Balme les Grottes, France). All the carbohydrates used in fermentation, growth assays and ATP measurements were purchased from Sigma-Aldrich or Carbosynth.

### Growth on carbohydrate substrates

For monitoring growth on different sugars *S. pneumoniae* cells were resuspended from agar plates in CAT (see above) without added carbohydrate at OD_590_ = 0.2, and diluted 1∶100 into fresh CAT medium with 200 U/µl of catalase and serial dilutions of sugars (usually 1%, 0.3% and 0.1%). Glucose served as positive control and medium with no added sugar served as a negative control. Microtiter plates were sealed with gas-permeable sealing membranes (Breath-Easy, Diversified Biotech, Boston, MA) and incubated at 37°C for 24 h in a thermostatic kinetic microplate reader (VERSAmax, Molecular Devices, Sunnyvale, Ca). OD_590_ was measured every ten minutes, and prior to each reading, plates were gently shaken for 2 s.

### Growth rate

Growth rate (μ) was established in the logarithmic growth phase calculating linear regression of the plots of ln(OD_590_) versus time, using at least five time points. The rate was then expressed as doubling time (time required to duplicate the bacterial biomass).

### Phenotype MicroArray analysis

Metabolism of *S. pneumoniae* strains DP1004 and rough derivatives of G54 and TIGR4 [105] were assayed by phenotype microarray (PM) microplates PM1 and PM2A (Biolog Inc, Hayward CA, USA) containing a total of 190 different carbon sources [134]. PM technology measures active metabolism by recording the irreversible reduction of tetrazolium violet to formazan as an indirect evidence for NADH production. PM procedures were mainly carried out as previously described [135]. Cells were grown overnight at 37°C on TSB agar plates (Tryptic Soy Agar, Beckton Dickinson) containing 200 U/ml catalase (Sigma). The cellular biomass was suspended in 15 ml of inoculation fluid (IF-0a GN/GP, Biolog, Inc.) containing Tween 85 and 5 mg/l choline (Sigma) using a sterile cotton swab, and the cell density was adjusted to 80% transmittance (T) on a Biolog turbidimeter. The 80% T suspension was diluted into inoculation fluid (1.6 ml in 24 ml) and 1% Redox Dye Mix G (Biolog, Inc.) was added. The mixture was inoculated in the PM plates (100 µl *per* well) followed by incubation at 30°C in an Omnilog Reader. Quantitative colour changes were recorded automatically every 15 min by a CCD camera for a period of 72 h. The kinetic responses of the strains was analyzed by the Omnilog-PM software (Biolog, inc.). The data were filtered using average height as a parameter. Strain DP1004 was analyzed in multiple runs and a consensus result was obtained. Rough derivatives of G54 were analysed in triplicate and those of TIGR4 once. The cut off for positivity was determined from graphs, where the frequency of log2 of the signal intensity (net average height = average height after incubation with cells – average height of medium only) was plotted. Unspecific colour development in the wells for pentoses and glucosamine did not allow detection of pneumococcal metabolism for these substrates.

## Supporting Information

Figure S1 **Schematic representation of pneumococcal PTS transporters.** All transporters shown are as in strain TIGR4, with the exception of an additional lactose type PTS (SPH1925-7,9) not present in TIGR4 (boxed). The five TC4.A.1 Glucose-Glucoside (Glc) PTS are shown in yellow, the three Fructose-Mannitol (Fru) PTS in pink, the TC4.A.3 Lactose-β-glucoside (Lac) PTS in red, the only TC4.A.5 Galactitol (Gat) PTS in light blue; the four TC4.A.6 Mannose-Fructose (Man) PTS in green and the two TC4.A.7 L-Ascorbate (L-Asc) PTS in blue. The different numbers of transmembrane segments of the transporters were deduced from TmPred predictions [137].
(TIF)

Table S1 **Primer list.**
(DOC)
