# Supplementary figures and images for: A Functional Genomics Approach to Establish the Complement of Carbohydrate Transporters in Streptococcus pneumoniae
Source: PLoS One. 2012 Mar 13;7(3):e33320. doi: 10.1371/journal.pone.0033320 (PMC3302838; doi:10.1371/journal.pone.0033320)

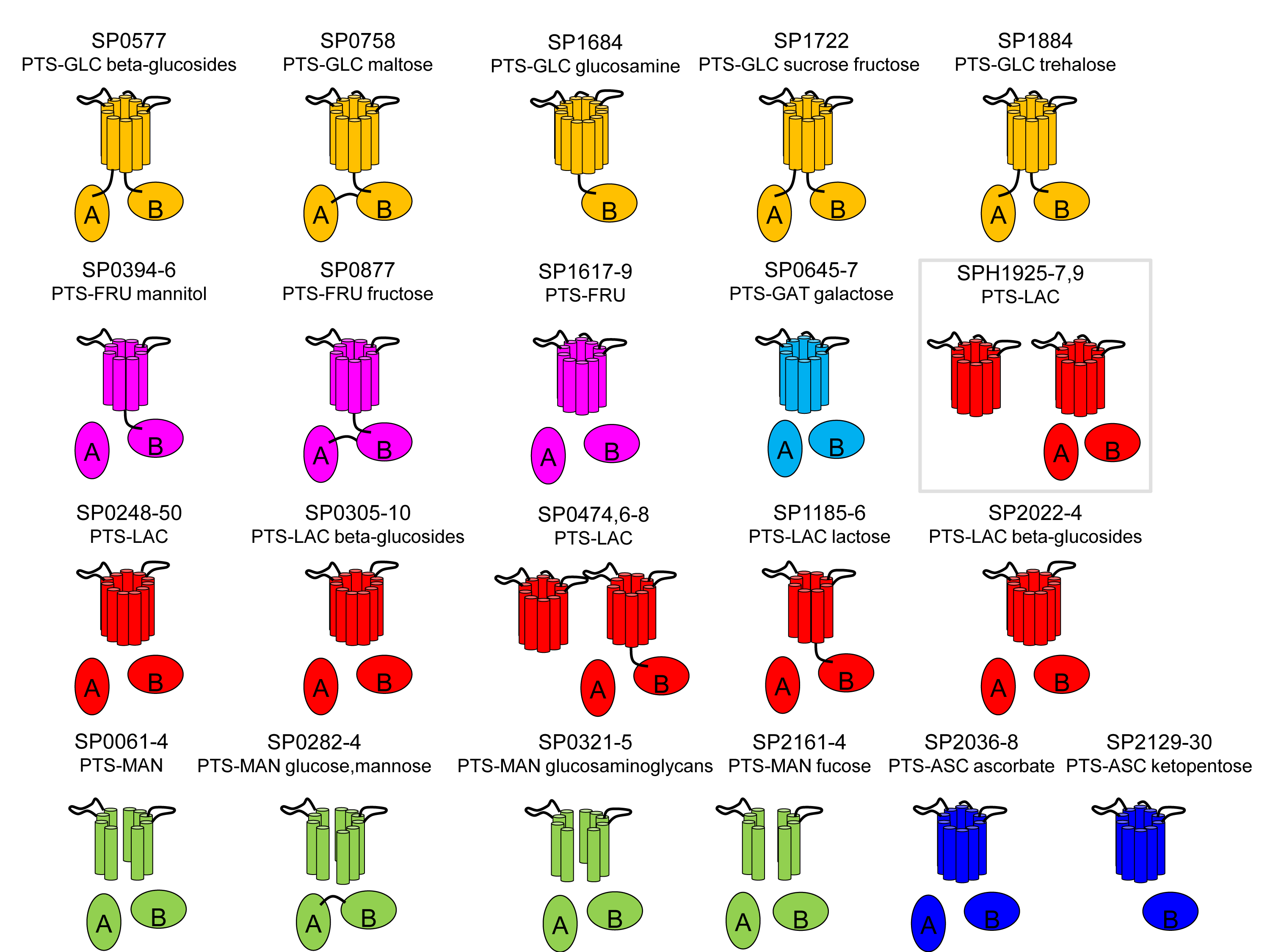

Supplement: Figure S1 — Schematic representation of pneumococcal PTS transporters. All transporters shown are as in strain TIGR4, with the exception of an additional lactose type PTS (SPH1925-7,9) not present in TIGR4 (boxed). The five TC4.A.1 Glucose-Glucoside (Glc) PTS are shown in yellow, the three Fructose-Mannitol (Fru) PTS in pink, the TC4.A.3 Lactose-β-glucoside (Lac) PTS in red, the only TC4.A.5 Galactitol (Gat) PTS in light blue; the four TC4.A.6 Mannose-Fructose (Man) PTS in green and the two TC4.A.7 L-Ascorbate (L-Asc) PTS in blue. The different numbers of transmembrane segments of the transporters were deduced from TmPred predictions [137]. (TIF) [file pone.0033320.s001.tif]
