# Supplementary material for: A Functional Genomics Approach to Establish the Complement of Carbohydrate Transporters in Streptococcus pneumoniae
Source: PLoS One. 2012 Mar 13;7(3):e33320. doi: 10.1371/journal.pone.0033320 (PMC3302838; doi:10.1371/journal.pone.0033320)
Supplement: Table S1 — Primer list. (DOC) [file pone.0033320.s002.doc]

Supporting Table I: Primer list (to be continued in supporting Table II)

| **Gene** | **Primer Name** | **Sequence** |
| --- | --- | --- |
| SP0061-4 | 62 Up a | CAACCGCATCATTATCT |
| SP0061-4 | Mut 62 up c | ATCAAACGGATCCCCAGCTTGCATCCTTGTCTACTACGT |
| SP0061-4 | Mut 62 up c | CCTCCGCGGAATTTGTATCTATTGAAGGATTGGATGAT |
| SP0061-4 | 62 Low c | TGACGTAGTTTGAAGAGAT |
| SP0248-50 | LM_109 | CTTACAGGAAAGAACGATAGCA |
| SP0248-50 | LM_110 | ATCAAACGGATCCCCAGCTTGTGTTTCGCATCACCAGCATATAA |
| SP0248-50 | LM_111 | CCTACGAGGAATTTGTATCGTACCTTTCATTCTTGTTCCTG |
| SP0248-50 | LM_112 | CTCTCTCAGCATCAATATAAGG |
| SP0282-4 | AB036 | CAGGATCTATGATCTTTGGTGA |
| SP0282-4 | AB037 | ATCAAACGGATCCCCAGCTTG TTTACCGTCTCCGATAACAGTT |
| SP0282-4 | AB038 | CCTACGAGGAATTTGTATC AGATATGTCTGGTGGTATCCTT |
| SP0282-4 | AB039 | GCAAGGATAATAGTGATTGGAG |
| SP0282-4 | AB118 | GATCCACTAGTTCTAGAGC TTTACCGTCTCCGATAACAGTT |
| SP0282-4 | AB119 | GTATCGCTCTTGAAGGGAA AGATATGTCTGGTGGTATCCTT |
| SP0323-5 | AB052 | ACTACTATTACCTAGAAGCCCT |
| SP0323-5 | AB053 | ATCAAACGGATCCCCAGCTTG TTTCTTTGATAGTGACACCACC |
| SP0323-5 | AB054 | CCTACGAGGAATTTGTATC GGATACAAACTAGGAACAACTG |
| SP0323-5 | AB055 | TGCCAAGACAATCACTATTCCA |
| SP0394 | 394 Up a | TAGTCCAGCCTTCATGTCA |
| SP0394 | mut 394 low a | ATCAAACGGATCCCCAGCTTGTTGACGAGGCTGGGC |
| SP0394 | mut 394 up c | CCTACGAGGAATTTGTATCACGAGAAGAACAATTAT |
| SP0394 | 394 Low c | ATCTTCTCTTACCAACTGA |
| SP0473-4 | 474 Up a | TAAGCCAAATCCAAGTAAT |
| SP0473-4 | mut 474 low a | ATCAAACGGATCCCCAGCTTGACCACTAACAGAAAGCGT |
| SP0473-4 | mut 474 up c | CCTACGAGGAATTTCTATCTCGGGTCTTTAAGA |
| SP0473-4 | 474 Low c | CAATCTTAGGTAGCCAAT |
| SP0478 | 478 Up a | ATTGAAGATGGTGATTTAG |
| SP0478 | mut 478 low a | ATCAAACGGATCCCCAGCTTGATTGACTCCTCTATCTC |
| SP0478 | mut 478 up c | CCTACGAGGAATTTGTATCTACAGATAGGCTTGGAT |
| SP0478 | 478 Low c | AGATACCCAGCATAAGA |
| SP0577 | AB040 | AATGTGGTGAACAGGTAGCAAT |
| SP0577 | AB041 | ATCAAACGGATCCCCAGCTTG ACCAAGACTTTCAACATACCTC |
| SP0577 | AB042 | CCTACGAGGAATTTGTATC ATTATCCCAAGTGAAGGTAAGG |
| SP0577 | AB043 | ACCATCTTCATTATATGCTCCC |
| SP0645-7 | AB044 | AGACTTCGTTATTGGGCGGTTA |
| SP0645-7 | AB045 | ATCAAACGGATCCCCAGCTTG TTCTTGACTATCCGCTTCTAAG |
| SP0645-7 | AB046 | CCTACGAGGAATTTGTATC GTATCATCGCTATGGGTGTTA |
| SP0645-7 | AB047 | TTTGCTGCGTACTCTTCGTTA |
| SP0758 | AB032 | GGTAAGGCTTTGATGGTAGTTA |
| SP0758 | AB033 | ATCAAACGGATCCCCAGCTTGAACCTGAGATAATCCCTACGAA |
| SP0758 | AB034 | CCTACGAGGAATTTGTATCCTTCGTGTAACTGTTAAAGATGC |
| SP0758 | AB035 | GTTTACCTTCAAGACTTACTGTG |
| SP0876-7 | 877 Up a | TATCTAGTCTGACAGCAGA |
| SP0876-7 | mut 877 low | ATCAAACGGATCCCCAGCTTGGCCGTTCCGCAAGCCAC |
| SP0876-7 | mut 877 up c | CCTACGAGGAATTTGTATCGAGACCGTCTAAAGCAG |
| SP0876-7 | 877 LOW C | ATATCGTAAACAGACCA |
| SP1185-6 | AB048 | GGATAACAAGATCAAACTCTGG |
| SP1185-6 | AB049 | ATCAAACGGATCCCCAGCTTG TGCTATCATTCATTCTTGCTGC |
| SP1185-6 | AB050 | CCTACGAGGAATTTGTATC GCACGAAGATAGATATTACGAG |
| SP1185-6 | AB051 | TTTGAAATCGTAGCCTATGCTG |
| SP1684 | DC_25 | CAAACGTGATTATCCACCTCA |
| SP1684 | DC_26 | ATCAAACGGATCCCCAGCTTGCACCACCAATCCCCAAAAG |
| SP1684 | DC_27 | ATATTTTACTGGATGAATTGTTTTAGAAAAAACAGAGGAGAGTGATGG |
| SP1684 | DC_28 | GTAGAATGGGGCAGAACTAATC |
| SP1884 | LM 32 | CTGTTGGTTGGTCTTGGATG |
| SP1884 | LM 33 | ATCAAACGGATCCCCAGCTTGCGCTACACGAATCTAATACTC |
| SP1884 | LM 34 | CCTACGAGGAATTTGTATCTGCCTCCGATTGCCTGAAG |
| SP1884 | LM 35 | TGGTTAGGTTGGATACAGGGA |

Supporting Table II: Primer list (continued from supporting Table I)

| **Gene** | **Primer Name** | **Sequence** |
| --- | --- | --- |
| SP2022 | LM_36 | GAGATGGCGTGTTATGGAGTCA |
| SP2022 | LM_37 | ATCAAACGGATCCCCAGCTTGGTATCCAAAACAGTGACTTC |
| SP2022 | LM_38 | CCTACGAGGAATTTGTATCCACTGTCATTCCCATCTGTCT |
| SP2022 | LM_39 | AACGATGACAAGGTGTGTTGC |
| SP2036-8 | 2036 upA | CACCAGCAAGAAGAGCATCA |
| SP2036-8 | LM_25 | ATCAAACGGATCCCCAGCTTG-TTGCATTAGACCATTCAG |
| SP2036-8 | LM_26 | CCTACGAGGAATTTGTATC-ACCGTTGACTTTGACCATT |
| SP2036-8 | 2036 lowC | TGTTGAATAAGATAAGAGACT |
| SP2129-31 | 2129 upA | GAGCCCGTTGTCATATCTA |
| SP2129-31 | LM_27 | ATCAAACGGATCCCCAGCTTG-TTGCGACTAACATCCGAT |
| SP2129-31 | LM_28 | CCTACGAGGAATTTGTATC -TTCACTATCAGGTATGGGA |
| SP2129-31 | 2129 lowC | TTAGTGAGTTGCTGGAATC |
| SP2161-5 | LM_129 | CAACTAGAATAACTCCAATAGCC |
| SP2161-5 | LM_114 | ATCAAACGGATCCCCAGCTTGAGGAGATAGTTTGTTCTGGCTGA |
| SP2161-5 | LM_115 | CCTACGAGGAATTTGTATCAACCGTGACAAAGCCACATTAT |
| SP2161-5 | LM_130 | CTTATGCTATTGTTGCTACAGG |
| SPG1701 | AB072 | AAAGTTACTTCCAGTAGCGGCT |
| SPG1701 | AB073 | GATCCACTAGTTCTAGAGC CAATCAACCAACCAACAATAGTC |
| SPG1701 | AB074 | GTATCGCTCTTGAAGGGAA AGCAATGTTTGGCGTTCCTATT |
| SPG1701 | AB075 | CAAATGTCTCACGAGTAACTAC |
| SP1176 | AB028 | ATTGTTGAGCATTCTGTAAGGG |
| SP1176 | AB029 | GATCCACTAGTTCTAGAGC ACACAAATCCGTGCTCTTCTT |
| SP1176 | AB030 | GTATCGCTCTTGAAGGGAA CCAAGAAGGTTTGCCAATACA |
| SP1176 | AB031 | CAGAAATGCTTAAAGGAATCGC |
| SP0090-2 | LM_117 | CATTGAGAGACAACTGGATCTT |
| SP0090-2 | LM_118 | ATCAAACGGATCCCCAGCTTG GAGTCAACTGAGGAATGGTTA |
| SP0090-2 | LM_119 | CCTACGAGGAATTTGTATC TTCGTGAAGAAACAGTAGGAC |
| SP0090-2 | LM_120 | AATCGCTTTATCTGGGTCTACA |
| SP0847-8 | AB086 | TGTCGGTGCCATTGAGTATAT |
| SP0847-8 | AB087 | AGATAGGCCTAATGACTGGCTTTTATAAAACCAAGACCAATCAAGACCA |
| SP0847-8 | AB088 | CCATTAAAAATCAAACAAATTTTCATAAAGTTTGGCTGTTATCGGTTC |
| SP0847-8 | AB089 | CTCTATTCCGTAACGATCAATC |
| SP1681-3 | DC_19 | CCGGTTCTATATGTTGTTCACG |
| SP1681-3 | DC_21 | TCCTGTCAAAATCTCTGTTCCATCCTACTCATCCATCACTCTC |
| SP1681-3 | DC_22 | CTATTATTTAACGGGAGGAAATAACCCTTGCTGCTGTTCCAATC |
| SP1681-3 | DC_23 | AAGCCAATGTCACTCGCTTC |
| SP1688-90 | DC_14 | AGCCGTTTAGGAACTTATGTG |
| SP1688-90 | DC_15 | GATCCACTAGTTCTAGAGCCGTTCTTCTCTTTCTACA |
| SP1688-90 | DC_16 | TTATAATTTTTTTAATCTGT |
| SP1688-90 | DC_18 | GGCGTCTCTTATTTCTACACC |
| SP1895-7 | AB003 | ACTATGGTAAATCTGCGGATG |
| SP1895-7 | AB004 | ATCAAACGGATCCCCAGCTTGATGGTGTTTGTCCTTTAGCAAC |
| SP1895-7 | AB009 | CCTACGAGGAATTTGTATCACGGTTATGATGAGTAAACTCG |
| SP1895-7 | AB006 | GAGATAGACAATGGTGATACTG |
| SP2108-9 | LM_125 | GAACTCACTGTATATGTAGACG |
| SP2108-9 | LM_126 | ATCAAACGGATCCCCAGCTTGTAGTAGAAGTTTGTCCAGTCAG |
| SP2108-9 | LM_127 | CCTACGAGGAATTTGTATCATGCTTACAGCCGTTACAACTT |
| SP2108-9 | LM_128 | ACCACTTGTAAGTCCTGAAACA |
| SP1580 | LM_139 | TTCTGACATTTGAACACCTCTG |
| SP1580 | LM_140 | ATCAAACGGATCCCCAGCTTGTCAGTTAATCTAGGGAGAGAAAC |
| SP1580 | LM_141 | CCTACGAGGAATTTGTATCGTCTTCTTTGCTGTATTTACGC |
| SP1580 | LM_142 | ACAGCGAACACTATTCAGTTGA |
| SP1580 | AB060 | GATCCACTAGTTCTAGAGCCGTATGATTACTGATTTCGTC |
| SP1580 | AB061 | GTATCGCTCTTGAAGGGAATCTTCTTTGCTGTATTTACGC |
| SP1307-39 | AB068 | TATCTGTGTATCTGAAGGTGC |
| SP1307-39 | AB069 | GATCCACTAGTTCTAGAGC GCTTCCTAGTTTGCTCTTTGAT |
| SP1307-39 | AB070 | GTATCGCTCTTGAAGGGAA GCATTCCATCAACTTCATCTGT |
| SP1307-39 | AB084 | TTATTGTCTATGTAGGAGCACC |
